# Xanthan gum derivatives: review of synthesis, properties and diverse applications

**DOI:** 10.1039/d0ra04366d

**Published:** 2020-07-21

**Authors:** Jwala Patel, Biswajit Maji, N. S. Hari Narayana Moorthy, Sabyasachi Maiti

**Affiliations:** Department of Pharmacy, Indira Gandhi National Tribal University Amarkantak Madhya Pradesh-484887 India sabya245@rediffmail.com +91 9474119931; Department of Chemistry, Indira Gandhi National Tribal University Amarkantak Madhya Pradesh-484887 India

## Abstract

Natural polysaccharides are well known for their biocompatibility, non-toxicity and biodegradability. These properties are also inherent to xanthan gum (XG), a microbial polysaccharide. This biomaterial has been extensively investigated as matrices for tablets, nanoparticles, microparticles, hydrogels, buccal/transdermal patches, tissue engineering scaffolds with different degrees of success. However, the native XG has its own limitations with regards to its susceptibility to microbial contamination, unusable viscosity, poor thermal and mechanical stability, and inadequate water solubility. Chemical modification can circumvent these limitations and tailor the properties of virgin XG to fulfill the unmet needs of drug delivery, tissue engineering, oil drilling and other applications. This review illustrates the process of chemical modification and/crosslinking of XG *via* etherification, esterification, acetalation, amidation, and oxidation. This review further describes the tailor-made properties of novel XG derivatives and their potential application in diverse fields. The physicomechanical modification and its impact on the properties of XG are also discussed. Overall, the recent developments on XG derivatives are very promising to progress further with polysaccharide research.

## Abstract 2

Due to presence of hydroxy and carboxy functional groups, xanthan gum is amenable to various chemical modification for producing derivatives such as carboxymethyl xanthan and carboxymethyl hydroxypropyl xanthan with desirable properties for end use.

## Graphical abstract

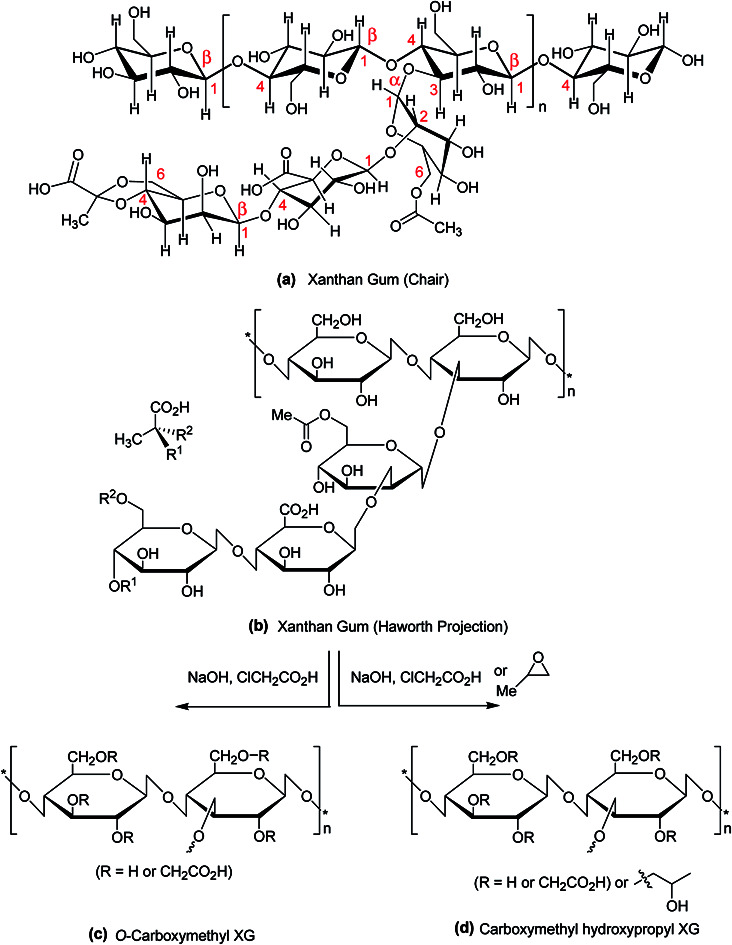

## 1. Introduction

**Fig. 1:**
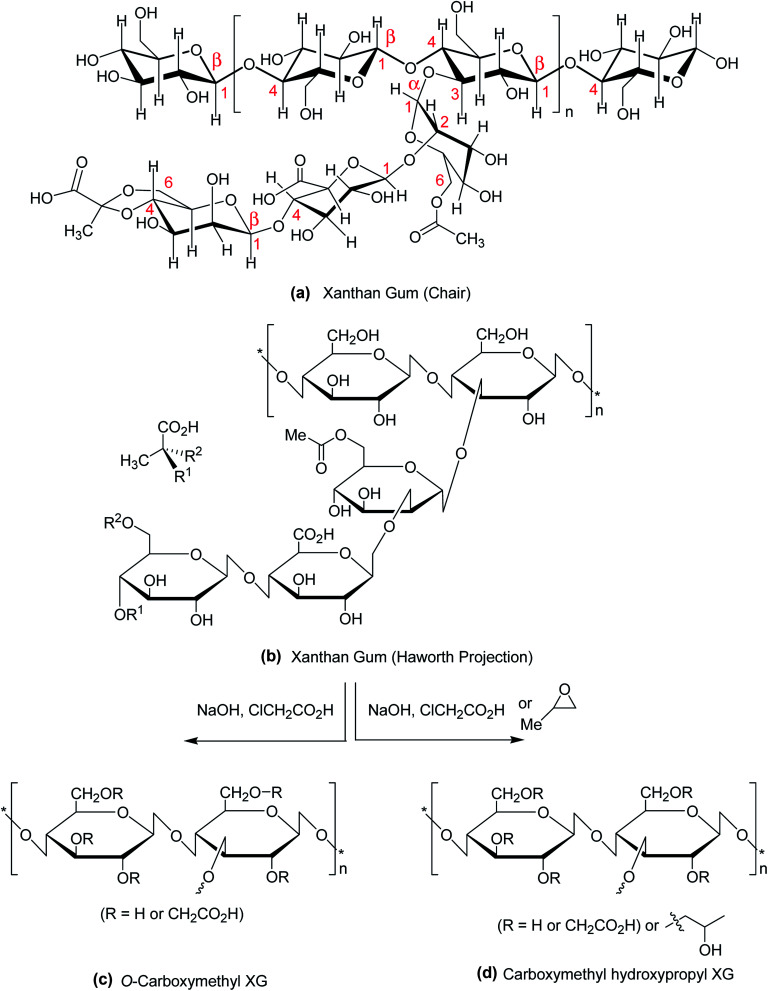
Chemical structure of (a) XG (chair conformation); (b) XG (Haworth projection); (c) carboxymethyl XG; (d) carboxymethyl hydroxypropyl XG.

Polysaccharides are isolated from renewable sources and have nontoxic, biocompatible, biodegradable, and bioadhesive properties. These qualities account for their use in food, pharmaceutical, biomedical and cosmetic applications. Among them, xanthan gum (XG) has attracted considerable interest in the past two decades due to its safety clearance by the US FDA as food additive in 1969.^1^ XG is a high molecular weight (2 × 10^6^ to 20 × 10^6^ Da) fermentation product of the Gram-negative bacterium *Xanthomonas campestris*.^2,3^ Chemically, it consists of β-1,4-d-glucopyranose glucan backbone with a pendant trisaccharide side chain, composed of mannose (β-1,4), glucuronic acid (β-1,2) and terminal mannose residues. The side chain is attached to alternate glucose residue in the backbone by α-1,3 linkages. In the side chain, the terminal mannose moiety is partially substituted with a pyruvate residue linked as an acetal to the 4- and 6-positions; the internal mannose sugar closest to the backbone is acetylated at C-6. The *O*-acetyl and pyruvate residues deprotonate at pH > 4.5, increase charge density along the xanthan chains, thus enabling XG for Ca^2+^ ion-mediated physical crosslinking.^4,5^ The presence of glucuronic acid in side chain gives birth to the polysaccharide a polyanionic character. Chemical structure of XG is illustrated in Fig. 1a and b.

The hydroxy and carboxy polar groups of XG reconnoiter intramolecular and intermolecular hydrogen bonding interactions in aqueous solution.^6^ Due to high molecular weight and hydrogen bonding interactions, an aqueous solution of XG exhibits high intrinsic viscosity at low concentration,^7^ and behaves like a pseudoplastic fluid.^8^ High viscous rheology and pH- and salt-resistant properties of XG are satisfactory for use as thickening agent, suspending agent,^9,10^ and stabilizers for food, pharmaceuticals, cosmetics^11–14^ and drag reducer in oil drilling.^15^ XG assumes disorder conformation under low ionic strength or high temperature;^16^ however, the XG present themselves either in single or double helix conformations under reverse condition.^17^ As a consequence of 3-D association of XG chains, its aqueous solution confers weak gel-like properties.^18^ However, XG never forms true gels at any concentration,^19^ attributable to its weak, non-covalent intermolecular interactions. In comparison to gels, hydrogels are crosslinked 3-D networks of hydrophilic polymers which swell in water or biological fluids but do not dissolve. Hydrogels possess some other interesting characteristics such as biocompatibility, softness which put them forward as carriers for drug or proteins delivery.^20^ Cross-linking of XG can be done either by physical or chemical methods.^21,22^ Physical cross-linking is a well-established method to fabricate XG-based hydrogels.^23^ In this method, the concentrated polysaccharide is cross-linked through hydrogen bonding,^24^ electrostatic ionic force,^25^ π–π stacking,^26^ hydrophobic interaction, or host–guest inclusion^27^ to obtain hydrogels. However, the physically-formed hydrogels suffer from poor mechanical strength, perhaps due to intermolecular secondary interactions, generally weaker than covalent bonds.^28^ Further, the lack of thermal stability, pH or salt stability on aging limits their applications. On heating or dilution, XG physical gels are easily solubilized in aqueous solution^29^ and thus, they are not suitable as controlled drug release carrier. In contrast, the coordination bonding between XG polymer chains and trivalent metal ions leads to stable gels *via* dimeric or polymeric ionic bridges.^30,31^

XG has also been put forward as a component of hydrogel preparations with other polymers such as alginate, locust bean gum, chitosan in the form of microparticles^32,33^ and nanoparticles.^34,35^ One fascinating example is the combination of XG with locust bean gum. In equal proportion, they can form a firm, thermoreversible gel in aqueous medium through synergistic interaction.^36^ This observation captivates this polymer combination for use as control drug release carriers.^37^

It is noteworthy that XG can act as a stabilizer for the synthesis of gold nanoparticles. The presence of mannose sugars in XG further assist in the delivery of chemotherapeutic agents/siRNA/shRNA to mannose receptor overexpressing cancer cells.^35^ Nonetheless, XG-stabilized silver nanoparticles have been studied for their antibacterial and catalytic applications.^38^ As a coating material, XG appears promising for the production of iron nanoparticles of 20–80 nm diameters.^39^ Recently, liposomes coated with chitosan–XG have been proposed as potential carriers for direct drug administration to lungs due to their improved liposomal stability and mucoadhesion properties.^40,41^

Native XG has been extensively studied as matrix forming materials in the design of tablets either alone^42–47^ or in combination with other natural polymers such as guar gum,^48^ alginate,^49,50^ cashew gum,^51^ konjac glucomannan,^52^*Gleditsia sinensis* Lam. galactomannan,^53^*Mimosa scabrella* galactomannan^54^ for the controlled delivery of various kinds of drugs. Chitosan and XG is an entrancing binary composition. The anionic gum electrostatically interacts with chitosan and forms polyelectrolyte complexes which serve as release-retardant for drugs.^55,56^ Even, the biocompatibility of chitosan–xanthan polyionic complex favors its pharmaceutical application.^57^

XG is not digested in human stomach or small intestine, but degraded in presence of colon enzymes.^58^ Thus, XG matrices can shield the drug from the environments of stomach and small intestine and deliver the drug to the colon. On reaching colon, they are assimilated by the anaerobic microflora of the colon, for example, bacteroides, bifidobacteria species and eubacteria, to smaller monosaccharides, which are then either used as energy source by the bacteria or degraded by enzyme. Keeping this in view, XG-based colon drug delivery systems are devised and tested for possible colon targeting of drugs. The systems are not limited to XG^59^ but include other polymers such as konjac glucomannan,^60^ chitosan/eudragit,^61^ guar gum^62^ as a component.

XG has an excellent biomimicking potential for tissue engineering applications such as bone, cartilage, and skin regeneration. XG-based hydrogels composed of other natural polymers and nanohydroxyapatite are fabricated and tested for tissue engineering applications^63,64^ and cellular uptake studies.^65^ In addition, the use of XG with electroactive polypyrrole has shown significant improvement in fibroblast proliferation.^66^ Kumar *et al.*^67^ found that XG-based hybrid scaffold system supported cell adhesion and proliferation of preosteoblast cells and thus seemed suitable for tissue engineering applications.

Its topical drug delivery application *via* intranasal, ocular, buccal and transdermal routes is also noteworthy. With large surface area, porous endothelial membrane and high total blood flow bypassing hepatic, gut wall metabolism and/or destruction in the gastrointestinal tract,^68^ the intranasal route appears to be a reliable alternative to oral and parenteral route for drug delivery. For example, XG-based *in situ* nasal gel has shown its unique ability to enhance drug permeation, bioavailability, and deliver drug directly to brain *via* olfactory lobe pathway.^69–72^ In context of ocular therapy, the major problem lies in attaining optimal drug concentration at the site of action. This is largely due to precorneal loss as a consequence of blinking of eyes.^73^*In situ* gel forming system appears to be the solution to this problem. The ocular bioavailability can be enhanced by increasing pre-corneal retention time. Accordingly, *in situ* ocular gels of XG in combination with other polymers such as poloxamer-407–guar gum,^74^ HPMC K4M,^75^ alginate–carbopol 934,^76^ gellan gum,^77,78^ alginate–ethylcellulose,^79^ poloxamer 407/188 (ref. 80) have been tested. Ion-induced nanoemulsion-based *in situ* gelling system of XG with HPMC or carbopol is also examined.^81^ Reports on *in situ* vaginal gels for eradication of candidiasis^82^ and *in situ* gel forming tablets^83^ are also evident. XG-based ophthalmic liquids,^84^ vaginal gels,^82,85^ bioadhesive liquids laden with lipid nanoparticles,^86^ microemulsion-based hydrogel^87^ and additives for spray-drying and freeze-drying processes^88,89^ are reported as well.

There is an expanse of smooth muscle and immobile mucosa in buccal cavity, which sets a platform for delivering drugs directly to the systemic circulation through the internal jugular vein, thus avoiding hepatic metabolism and acid hydrolysis in the gastrointestinal tract.^90^ Moreover, rapid mucosal cell recovery is attractive for opting buccal route for drug delivery purpose.^91^ These inspired researchers to develop nicotine patches for smoking cessation,^92^ desloratidine patches for treating allergic rhinitis^93^ and zolmitriptan patches for migraine treatment.^94^ Transdermal patches further provide benefits in terms of reduced first-pass metabolism, frequency of dosing, side effects secondary to gastrointestinal intolerance and fluctuations in drug levels.^95^ Transdermal films/patches of XG with other polymers such as alginate,^96,97^ hydroxypropylmethyl cellulose–carboxymethyl cellulose^98^ are reported for analgesic and antihypertensive drugs.

The beneficial effects after oral administration of XG on immune-surveillance against neoplasms are also reported.^99^ Low-molecular-weight XG (4.07 × 10^4^ Da) has shown antioxidant and excellent protective effect on H_2_O_2_-injured Caco-2 cells, suggesting its use in food/pharmaceuticals in order to alleviate/resist oxidative damage induced by overproduction of reactive oxygen species.^100^ XG is a strong inhibitor of oil peroxidation^101^ and antioxidant for human corneal epithelial cells at 0.2% strength.^102^

Despite numerous potential benefits, native XG poses some problems which include microbial contamination, unstable viscosity, poor shear resistance, inadequate mechanical, thermal properties and uncontrolled rate of hydration. These limit its applications in food, pharmaceutical and biomedical fields. Further, XG dissolves at a slow rate particularly in cold water at high concentration. Upon dispersion, it forms lumps in water called fish eyes^103^ due to its improper hydration. Consequently, a gelatinous outer layer is formed on surface of the particles, immediately after dispersion. This gelatinous layer prohibits water penetration and hinders complete dissolution of the particles.^104^ Due to insufficient gelling, XG itself fails to afford self-sustainable, stable hydrogel beads in presence of metal ions. XG possesses a number of hydroxy and carboxy groups which are amenable for chemical modification with successive improvement in its physicochemical properties especially solubility, swelling and metal-induced gelling ability; mechanical and thermal stability. The chemical derivatives then become widely acceptable for diverse applications. As extracted from scientific literatures, the chemical modification of XG involves etherification, esterification, acetalation, oxidation, peptide linking, ionic and covalent crosslinking, and mechanical modification. However, there are no decent reviews that sum up the data on modification procedures of XG, resultant properties and the diverse application of XG derivatives.

Though some reviews cover industrial production,^105–107^ and drug delivery and biomedical applications of XG,^108–111^ these are not adequate. In 2003, Badwaik *et al.*^112^ published a review on XG and its derivatives which discussed the synthesis of acetalated and carboxymethyl derivatives of XG, microwave and plasma assisted grafting of XG. The authors mostly focused on drug delivery application. However, this review lacks significant, in-depth and detailed up-to-date information on synthetic procedures, properties and diverse applications of modified xanthan polysaccharide. Till date, no review article describes XG derivatives in a comprehensive manner in terms of their detailed synthetic/crosslinking procedures, tailored properties beneficial for industrial applications in the field of pharmaceutical and biomedical science and technology. This review attempts to cover these aspects of XG derivatives to fulfill the unmet needs of students, researchers, and budding scientists in these fields.

## 2. Modified xanthan materials

### 2.1. Etherified XG

The most common and widely investigated ether derivatives of XG is the *O*-carboxymethyl XG. Carboxymethylation is a simple chemical etherification reaction which requires simple chemicals like monochloroacetic acid and sodium hydroxide (Fig. 1c). The degree of *O*-carboxymethyl substitution may vary proportionately with chloroacetic acid : XG ratio within 0.5 to 3.^113^ The viscosity, elastic modulus and molecular weight of XG are lessened after modification. The derivative becomes more hydrophilic than native XG with gradual increase in degree of substitution. However, the shear-thinning and weak gel behaviors of XG are not affected after carboxymethylation. In contrast, an aqueous solution of carboxymethyl hydroxypropyl XG (Fig. 1d) exhibits greater viscosity, elastic modulus, and temperature- and shear-resistance than XG solution at the same strength. A better proppant carrying ability makes this derivative suitable for use as an additive of fracturing fluids.^114^

**Fig. 2:**
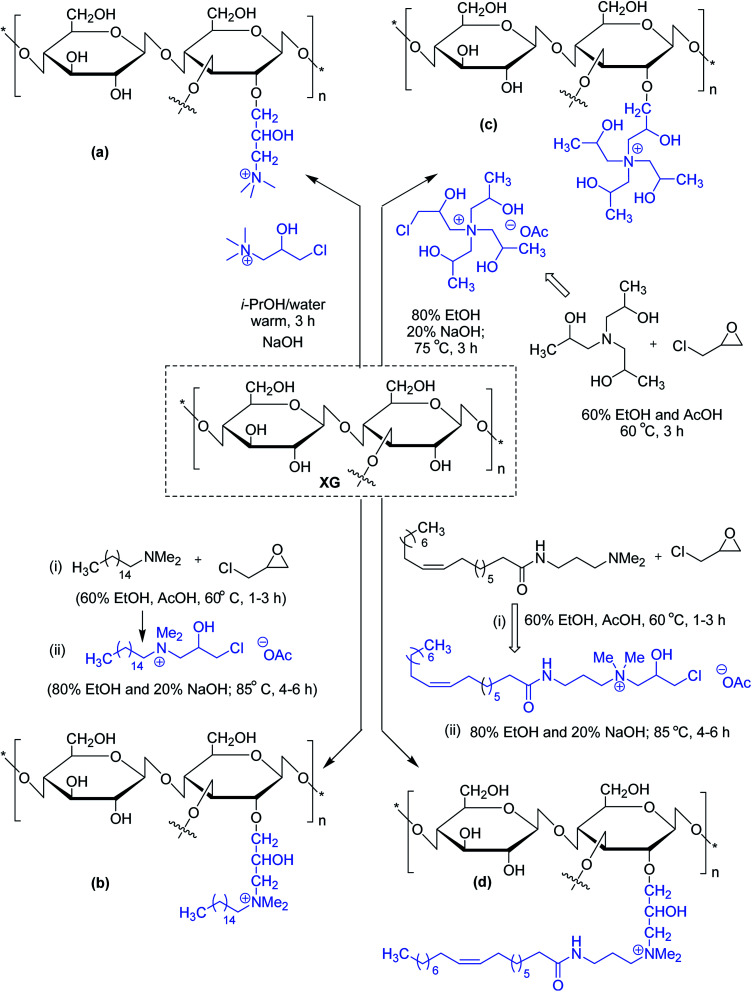
Synthesis of cationic and amphoteric XG derivatives.

Similar viscoelastic behaviors are noticed for cationic and amphoteric XG derivatives, synthesized using *N*-(3-chloro-2-hydroxypropyl)trimethyl ammonium chloride^115,116^ and 3-chloro-2-hydroxypropyldimethylhexadecylammonium acetate,^117^ respectively. The amphoteric XG solution appears to be a better drag reducer than virgin XG solution. Oleamidopropyl dimethylamine- and triisopropanolamine-modified amphoteric derivatives of xanthan have been reported for their high viscosity (Fig. 2).^118^

**Fig. 3:**
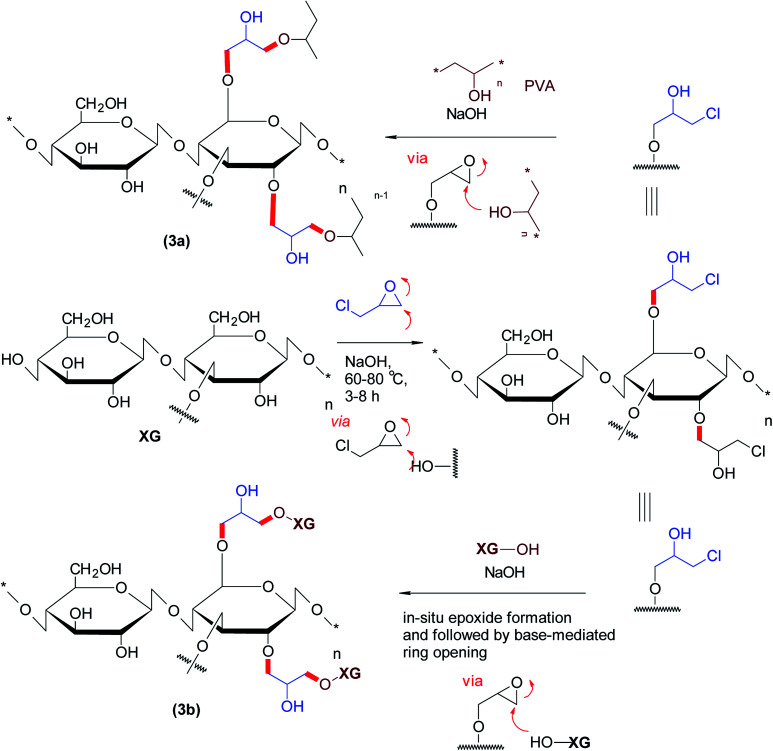
Epichlorohydrin-crosslinked XG or PVA/XG.

The etherified derivatives of XG have been assessed for their quality attributes in the form of hydrogel films. Alupei *et al.*^119^ synthesized epichlorohydrin-crosslinked poly (vinyl alcohol) (PVA)/XG supersorbent hydrogel films (1 mm thickness) under basic medium and appraised their controlled swelling *in vitro*. Interestingly, higher polymer : crosslinker ratio (5 : 1) allowed swelling of the hydrogels up to ∼2500%. Dhar and collaborators^120^ highlighted the importance of pH on crosslinking reaction of epichlorohydrin with PVA/XG. They perceived that labile ester linkages predominated in the composite films at pH < 4; whereas both ether and ester bonds were registered over pH 7.0–7.5 and consequently, manifested different degrees of swelling in water. They argued that the films synthesized at pH > 9 were mechanically strong due to ether linkages and thus swelled progressively. Incorporation of XG in PVA films caused lesser swelling of the composite films and proved useful in controlling release of uric acid over 24 h at pH 7.4 (Fig. 3).

Zhang *et al.*^121^ spotted highly viscous, thixotropic, viscoelastic and thermoresistant characteristics of XG after crosslinking under alkaline condition at epichlorohydrin : XG weight ratio of 4 : 200, beyond which these additive effects nullified. The crosslinking effect of epichlorohydrin on theophylline loading and release from xanthan/chondroitin sulfate (1 : 1) hydrogels was assessed by Oprea *et al.*^122^ They noted that about 78% theophyline loading was possible by diffusion filling method. The hydrogels swelled within a minute but thereafter absorbed simulated gastric and intestinal fluids at a slower rate. The inclusion of chondroitin sulfate in the hydrogels suppressed drug release by 34% in acidic fluid (pH 2.2) in 7 h. However, they spotted a reverse phenomenon in intestinal fluid (pH 7.4). An enhanced oral bioavailability of 323% clearly provided an indication of preclinical success of this hydrogel system.

Maiti and groups^123^ proposed hexadecyl etherification of XG as a means of enhancing solubility of glibenclamide through polymeric micellization process. The copolymer provided 122-fold higher aqueous solubility than pristine drug. The micelles had a command on the drug release in simulated biological fluids up to 8 h and on diabetes in alloxan-induced hyperglycemic rabbit model. The enmeshing of drug-loaded copolymer micelles into *O*-carboxymethyl aluminium xanthan hydrogel particles declined drug release rate in simulated fluids. They discerned better hypoglycemic activity of glibenclamide after trapping micelles into carboxymethyl XG hydrogels. The glucose-lowering action lasted up to 8 h in alloxan-induced hyperglycemic rabbits.^124^ Quan *et al.*^125^ conjugated hexdecyl groups to XG under its ordered conformation, where its structure remains altered. Intriguing to note that, this derivative associated at a very low concentration (0.16%) in water, and showed better thermal resistance, and 4-fold higher viscosity. Altogether the hydrophobic modification of XG under order conformation contributed significantly to these changes in XG properties.^125^

**Fig. 4:**
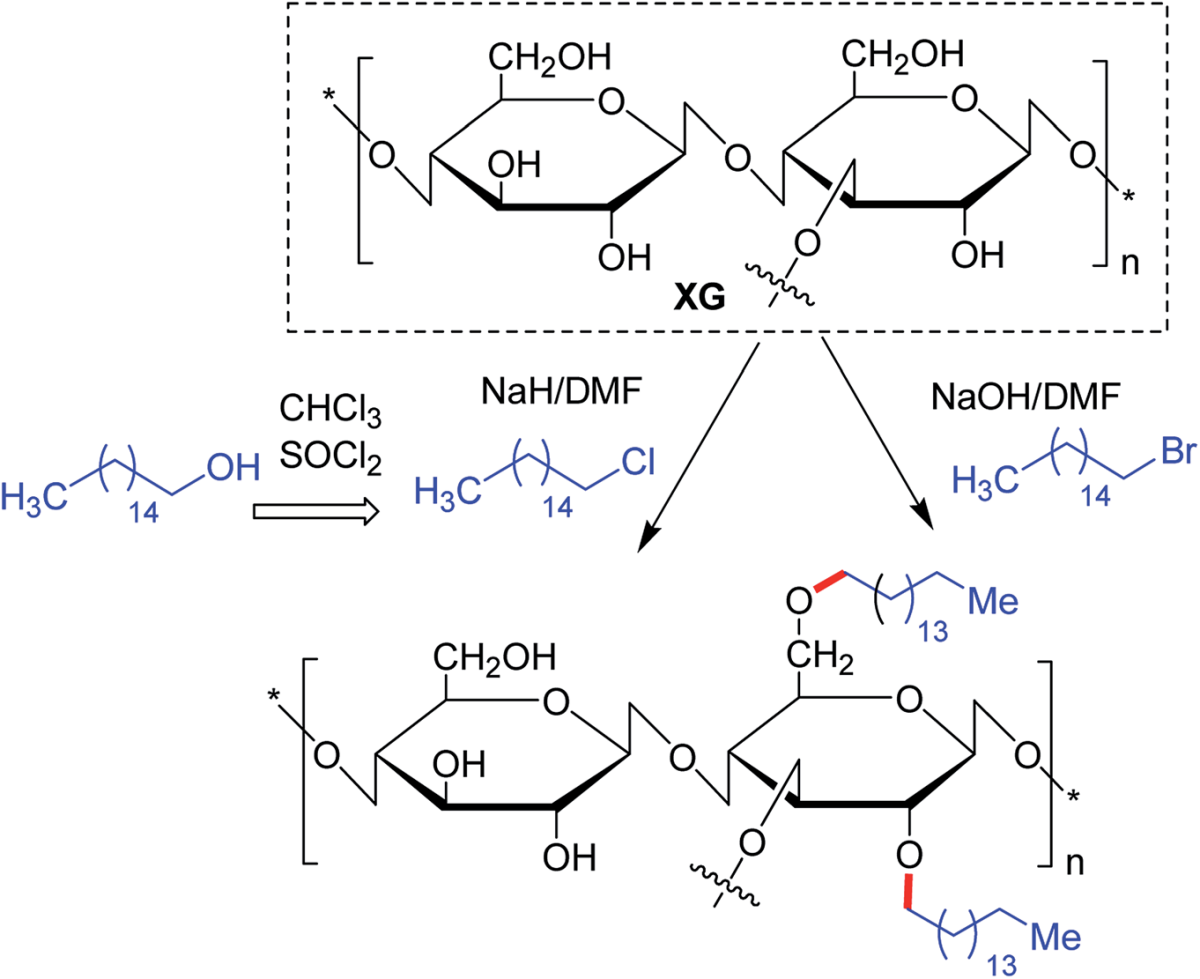
Long alkyl chain conjugated XG derivatives.

The substitution of 4- or 8-tetradecyl chains to XG assisted in denser association of the polymer network, resulting in higher solution viscosity. Greater hydrophobic interaction amongst the polymer chains was presumed for this close association at higher polymer concentration and alkyl substitution, though the temperature had a negative impact^126^ (Fig. 4).

Bakshi *et al.*^127^ found that solid dispersion prepared by hot melt process in combination with microwave irradiation resulted in 75-fold increase in glibenclamide solubility and better preclinical hypoglycemic activity at a drug : Soluplus® ratio of 1 : 7. This observation invigorated them to incorporate the solid dispersion into carboxymethyl XG mini-matrices in order to circumvent the fluctuations in dissolution rate associated with pure drug under gastrointestinal fluids. Carboxymethyl XG matrices containing Soluplus gradually released the drug up to 70% till the end of 8 h without any burst effect. On contrary, XG matrices showed burst release of drug and corresponded to 100% in 6 h. It was noteworthy that the solubility enhancement caused by Soluplus dispersion was about 50-fold lower than that reported for hexadecyl xanthan copolymer.^127^

Ahuja *et al.*^128^ proceeded with synthesis and physical characterization of the carboxymethyl XG. They reported globular shape morphology and crystalline nature of carboxymethyl XG particles. The viscosity deteriorated after carboxymethylation. Carboxymethyl XG matrices revealed faster release of diclofenac sodium compared to unmodified XG. For brevity, the carboxymethyl XG matrices emptied its entire content in 3 h; while unmodified XG released half of its content in 16 h. It was postulated that less viscous carboxymethyl XG promoted penetration of water into the matrix, caused faster dissolution of the matrix resulting in faster drug release in phosphate buffer solution (pH 6.8). However, the release profiles of diclofenac-loaded carboxymethyl XG matrices were contradictory to that reported by Bakshi *et al.*^127^

Munir *et al.*^129^ reported better thermal stability and DPPH free radical scavenging (antioxidant) activity of XG after carboxymethylation. The antibacterial effects of gum became intensified after modification. In another study, carboxymethyl XG capped gold nanoparticles showed their unique excellence in cancer cell killing. Microwave synthesis of carboxymethyl XG-capped gold nanoparticles containing doxorubicin exhibited about 4.6-folds higher anticancer efficacy *in vitro* in presence of an ionophore (*i.e.* nigericin) than free doxorubicin.^130^

The *O*-carboxymethyl ether derivative of XG was largely synthesized for the fabrication of ionically and/covalently crosslinked hydrogel particles and tested for controlled drug delivery. The outcomes of such investigations are summarized in Table 1.

**Table 1:** Properties of carriers systems made with ether derivatives *via* ionic crosslinking, polyelectrolyte complexation, covalent cross-linking or both

Type of devices	Polymers/cross linkers	Research outcomes	References
PEC hydrogels	Chitosan and XG/Opadry	• Incorporation of metronidazole into pre-formed hydrogels in significant amount is possible by diffusion technique	131
		• Hydrogels swell less in simulated gastric medium	
		• Enteric coating allowed 50% drug for release in colonic pH	
PEC hydrogels	*N*-Trimehtyl chitosan (TMC)/carboxymethyl xanthan gum (CMXG)	• Encapsulation efficiency of ciprofloxacin reached to about 93.8% at higher drug load	132
		• Drug-loaded hydrogel was highly effective against the Gram positive and Gram negative bacterial strains	
		• Highest diameter of inhibition zone against *Escherichia coli* as compared to gentamicin	
		• Highest cell viability (97%) in lung human normal cell lines was noted at concentration up to 50 μg mL^−1^	
Ionic and polyelectrolyte complexation	Polyethyleneimine (PEI)/CMXG/AlCl_3_	• Increasing PEI (0.5–2%) and exposure time (5–30 min) decreased drug entrapment efficiency from 96.50 to 77.50% and from 92.25 to 70.37%, respectively	133
		• PEI treatment reduced swelling of the beads	
		• Depending on formulation variables, 40% and 80% drug released in 2 h in pH 1.2 and in 5–6 h in pH 6.8, respectively	
		• PEI treated diltiazem–resin complex beads released the drug following non-Fickian transport mechanism	
		• Bioavailability was 1.59 times higher with PEI-treated formulation than pure drug solution in rabbit model	
		• Data showed good *in vitro*–*in vivo* (IVIVC) correlation	
Magnetically responsive polyelectrolyte complex hydro-gels	XG and chitosan in the presence of iron oxide magnetic nanoparticles (MNPs) using d-(+)-glucuronic acid δ-lactone as a green acidifying agent	• Incorporation of Fe_3_O_4_ MNPs (8 nm size) into complex hydrogels induced porosity and greatly improved mechanical properties and storage modulus	134
		• Magnetically responsive hydrogels improved NIH3T3 fibroblasts cell proliferation and adhesion in an external magnetic field relative to pristine hydrogels without MNPs *in vitro*	
		• Suitable for use as a magnetically stimulated system in tissue engineering applications	
Tablets	Polyethylene glycol/XG/chitosan	• Drug release profiles of tested metronidazole tablets and commercial ER formulation were similar in 0.1 M HCl and phosphate buffer pH 6.8	135
		• Bioadhesion of tested tablets was three times higher than commercial tablets to sheep duodenum	
		• Absorption of metronidazole from test product was faster than that of commercial product with a maximum plasma level attained at 4.37 and 6.14 h, respectively	
Interenetrating network (IPN) hydrogel beads	CMXG and carboxymethyl cellulose/AlCl_3_	• Methacrylic acid-based ion exchange resins (IER) were synthesized using ethylene glycol dimethacrylate, *N*,*N*′-methylene bis acrylamide, and divinyl benzene coded as ME, MB and MD respectively	136
		• IER : ofloxacin ratio of 1 : 2 provided highest drug loading ∼98% MD and MB; however, the same was ∼85% for ME	
		• Taste masking studies at salivary pH 6.8 showed that MD : ofloxacin (1 : 4) showed lowest (1.22%) release of drug for a contact time of 30 s than others	
		• Presence of bulky divinylbenzene imparted steric hindrance for the exchange of phosphate groups with the amine groups present in drug at salivary pH, ultimately resulting in slow release of the drug	
		• CMXG beads containing MD : ofloxacin (1 : 4) (636 μm) had highest drug entrapment efficiency of 91.90% following treatment with 2% AlCl_3_	
		• MD : ofloxacin (1 : 4)-carboxymethyl XG/carboxymethyl cellulose IPN beads (1122 μm) had 90.23% drug entrapment efficiency	
		• Compared to gastric pH, drug release from MD : ofloxacin (1 : 4)-carboxymethyl XG beads and carboxymethyl XG/carboxymethyl cellulose IPN beads at intestinal pH 7.4 became prolonged and extended up to 10 h	
IPN hydrogel beads	Casein and CMXG/aluminium chloride/glutaraldehyde	• Glutaraldehyde treatment prohibited extent of degradation of hydrogels in pH 7.4 phosphate buffer containing 0.2% lysozyme	137
		• Theophylline release was slower in pH 1.2 buffer solution than in pH 6.8 buffer	
		• Increasing carboxymethyl XG : casein ratio decreased the extent of drug release marginally both in acidic media and alkaline media in 2 h	
		• Higher carboxymethyl XG : casein ratio and drug loading improved drug entrapment efficiency from 28.6 to 53.81% and from 53.81% to 83.59%, respectively	
		• Increase in glutaraldehyde concentration caused lowering of drug entrapment efficiency from 83.01 to 40.03% upon 15 min exposure. Reduction of exposure time to 5 min increased DEE to 85.12%	
		• Theophylline transformed into amorphous state after entrapment	
		• Shifting of pH of dissolution medium from 1.2 to 6.8 caused significant swelling of beads	
		• Increase in AlCl_3_ concentration (2–8%) increased swelling of beads by 10.26% and 11.62% in pH 1.2 buffer and pH 6.8 buffer solutions, respectively	
IPN hydrogel beads	Pectin/CMXG/Al^3+^ ions and covalent cross-linking with glutaraldehyde	• Both swelling and drug release were relatively low in pacidic medium than in pH 6.8 buffer	138
		• *In vitro* delivery of diltiazem was dependent upon the extent of cross-linking and amount of drug used in the IPN hydrogel beads	
IPN hydrogel beads	CMXG and sodium alginate/AlCl_3_	• *C*_max_ was significantly less and *T*_max_ (2.91 ± 0.5) was relatively higher from the drug loaded IPN beads than those from the control and reference in rabbit model	139
		• Relative bioavailability of test formulation was 109.02% and 112.79% compared to control and reference, respectively	
Compression coated tablets	CMXG and sodium alginate/CaCl_2_	• Compression-coated tablets of Ca^2+^ ion crosslinked CMXG and sodium alginate could deliver prednisolone without the need of colonic bacterial intervention for degradation of the polysaccharide coat	140
		• Blend of CMXG and alginate (1.5 : 3.5) provided *T*_lag_ of 5.12 h and *T*_rap_ (time required for immediate release following *T*_lag_) of 6.50 h	
Hydrogel beads	CMXG/AlCl_3_/glutaraldehyde	• Diltiazem–cation exchange resin was loaded into carboxymethyl XG hydrogel beads	141
		• Sequential cross-linking involving glutaraldehyde treatment of ionically preformed hydrogel beads produced smaller beads with higher drug entrapment efficacy (86.52%) and prolonged release characteristics than ionic cross-linking, and simultaneous ionic/covalent cross-linking	
		• Swelling of the beads was higher in acid solution of pH 1.2 than in buffer solution of pH 6.8	
		• Burst release (∼50%) in acid solution, followed by extended release up to about 7 h	
Tablets	CMXG/CaCl_2_	• Increase in degree of calcium co-ordination/cross-linking reduced swelling of Ca–CMXG matrix tablets compared with carboxymethyl XG matrix	142
		• Erosion of Ca–CMXG matrices was higher than CMXG matrix	
		• Release of prednisolone from Ca–CMXG matrices containing upto 33.33% (w/w) CaCl_2_ was less than that from CMXG matrix	
		• Release of drug from the matrix containing 50% (w/w) of CaCl_2_ was rapid and approached almost to that from CMXG matrix	
IPN beads	Carboxymethyl cellulose and CMXG/AlCl_3_	• Higher extent of cross-linking led to decreased particle size from 1080 to 1420 μm	143
		• Variation in cellulose to gum ratio from 1 : 1 to 2 : 1 dropped drug encapsulation efficiency of IPN beads from 96.96 to 77.45%	
		• Drug entrapment efficiency of the beads decreased at higher salt strength	
		• Increase in salt strength from 4 to 8% slowed drug release rate in acidic and alkaline media	
		• Drug release continued up to 8 h in pH 7.4, indicating better intestinal drug release	
Homopolymeric and IPN beads	CMXG and sodium alginate/AlCl_3_	• Entire ibuprofen can be loaded into the beads with a maximum coefficient variation of 1.87%	144
		• IPN hydrogel beads provided more sustained release of ibuprofen than homopolymeric beads	
		• Rapid drug release from Al–CMXG beads, accounting 42.5% release in 2 h	
		• Incorporation of higher amounts of alginate in IPN beads decreased drug release	
		• Release of drug from Al–alginate beads was the lowest, releasing 25.4% drug in 2 h in acidic medium	
		• Release of drug was the fastest from Al–CMXG in same duration	
		• Drug release from Al–alginate beads was faster than those from the IPN beads in pH 6.8 phosphate buffer	
IPN beads	CMXG and sodium alginate/aluminium chloride	• Ulcerogenicity decreased significantly with ibuprofen-loaded IPN beads in comparison to the pure drug in adult male albino Wistar rats	145
Microparticles	CMXG/aluminium chloride	• CMXG or alginate-coated Al–CMXG microparticles were prepared	146
		• Higher salt concentration decreased BSA entrapment efficiency of the uncoated microparticles from 86–61%	
		• BSA entrapment in coated microparticles was found lower (78–79%) than uncoated microparticles	
		• Uncoated microparticles released almost half of its content in NaCl–HCl buffer solution (pH 1.2) in 2 h	
		• Alginate and xanthan coated microparticles did not liberate a substantial amount of entrapped protein in acidic medium, rather prolonged protein release in PBS solution (pH 7.4) up to 10 and 12 h, respectively	
		• Sodium dodecyl sulfate–polyacrylamide gel electrophoresis indicated retention of protein integrity in the microparticles	
Microparticles	CMXG/aluminium chloride	• Variation in pH of carboxymethyl XG solution did not affect protein entrapment and release significantly	147
		• Increase in initial protein loading tended to increase protein release in buffer solution of pH 1.2 and in PBS solution (pH 7.4)	
		• Higher polymer concentration suppressed protein release substantially in both acidic and alkaline media	
		• Maximum 86.39% protein entrapment efficiency was noted	
Hydrogel beads	CMXG/aluminium chloride	• Diltiazem–resin complex was loaded into CMXG beads	148
		• Higher gelation period (5–20 min) and AlCl_3_ concentration (1–3%) decreased drug entrapment efficiency from 95 to 79% and 88.5 to 84.6%, respectively	
		• Gum concentration up to 2.5% improved drug entrapment efficiency to 90.7%	
		• Higher swelling was accounted for faster drug release in simulated gastric fluid than in intestinal fluid	
Hydrogel beads	CMXG/aluminium chloride	• Viscosity of CMXG was always lower than that of XG	149
		• Formation of discrete and spherical BSA-loaded microparticles were dependent on microenvironmental pH	
		• BSA entrapment efficiency was 82%	
		• Higher swelling contributed faster protein release in acidic medium than that in alkaline medium	
		• pH of the gum solution influenced the swelling and protein release considerably	
Nanoparticles	XG/sorbitan monooleate/oleylamine	• XG-functionalised sorbitan monooleate/oleylamine nanoparticles had average diameter of 146.8 nm and high surface charge (−48 mV)	150
		• Core–shell morphology of enhanced green fluorescent protein plasmid (pEGFP) loaded nanoparticles was evident	
		• Cytotoxicity and transfection capacity of nanoparticles were excellent in human umbilical vein endothelial cells (HUVECs)	
		• Pre-clinical study confirmed the potential of XG-functionalized span nanoparticles for gene targeting to endothelial cells	
		• Xanthan shell protected associated DNA from DNase degradation, a prerequisite for intact delivery of bioactives to its site of action	
Layered Fe–XG hydrogels	XG/FeCl_3_	• Fe^3+^-coordination enabled XG to form hydrogels under ambient temperature	151
		• Fe–XG hydrogel exhibited a regular laminated structure under scanning electron microscope	
		• XG hydrogels possessed uniform layered structure, enhanced mechanical strength and excellent swelling behavior	
		• Sol–gel conversion of XG-based hydrogel could be realized by UV light in the presence of sodium lactate	
		• Sol–gel conversion ability of Fe–XG hydrogel could provide data for using as sensor to detect oxidizing or reducing agents, as an actuator under UV light with enough sodium lactate, or for the drug release in the future study	
Floating hydrogel beads	Chitosan/XG	• Variation in chitosan to XG ratio did not affect glipizide release behaviors of the beads in phosphate buffer pH 7.4 up to 24 h	152
		• Drug entrapment efficiency was 80–95%	
		• Beads possessed comparable buoyancy in gastric fluids and satisfactory bioadhesive strength	
		• Swelling kinetics differed significantly in pH 1.2 and 7.4 buffer	
Hydrogel beads	CMXG/carboxymethyl cellulose/AlCl_3_	• Beads released considerably less amount of aceclofenac in acid solution (maximum 14.2%) and provided controlled release in phosphate buffer solution (pH 6.8)	153
		• Aceclofenac was compatible with the matrix	
		• Morphology, size and drug entrapment efficiency of beads, and *in vitro* drug release was affected by viscosity of polymer dispersion, initial drug load, and concentration of total polymer and AlCl_3_	

### 2.2. Esterified XG

**Fig. 5:**
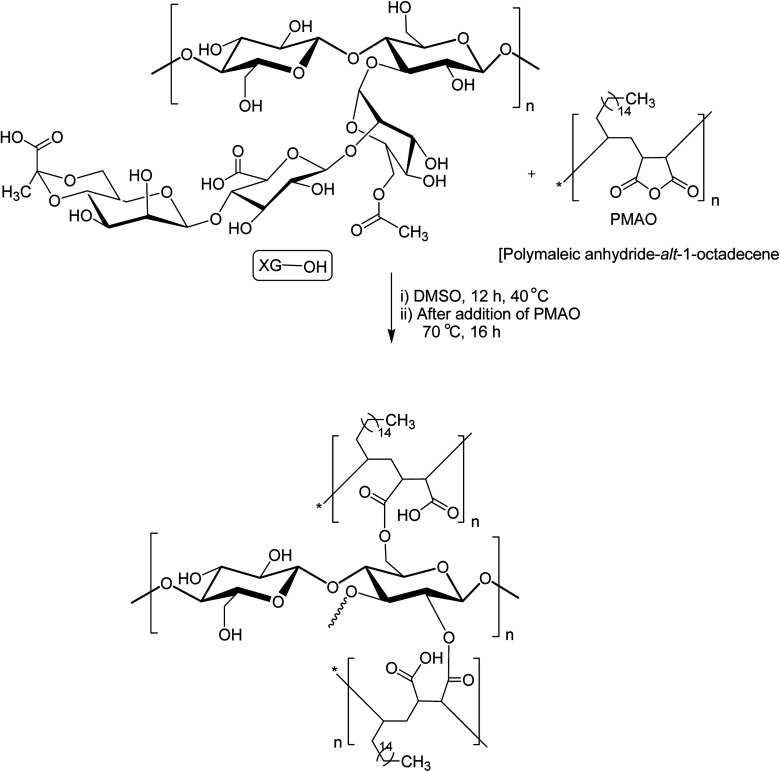
Esterification of XG with PMAO.

XG was also reshaped *via* esterification, and the derived properties were scrutinized for various applications. Qian *et al.*^154^ esterified the carboxy groups of XG by 1-bromooctane to obtain water soluble XG. They attained an octyl substitution of 11 and 21 per 100 structural units. The esterification promoted hydrophobic association and consequently enhanced the viscosity of XG. The deacetylated XG (∼1.3–1.4%) exhibited viscosifying effect^155^ similar to that observed after octyl substitution.^154^ When esterified with poly(maleic anhydride/1-octadecene) (PMAO) in dimethyl sulfoxide (DMSO) solution (Fig. 5), the modified XG offered an outstanding resistance to shear force and displayed viscoelastic behaviors, attributable to hydrophobic association of the polar anhydride group and non-polar C16 side chain. The authors further acknowledged its excellent salt tolerance as well as temperature resistance properties which could be beneficial in oil recovery, pharmaceutical and food applications.^156^

**Fig. 6:**
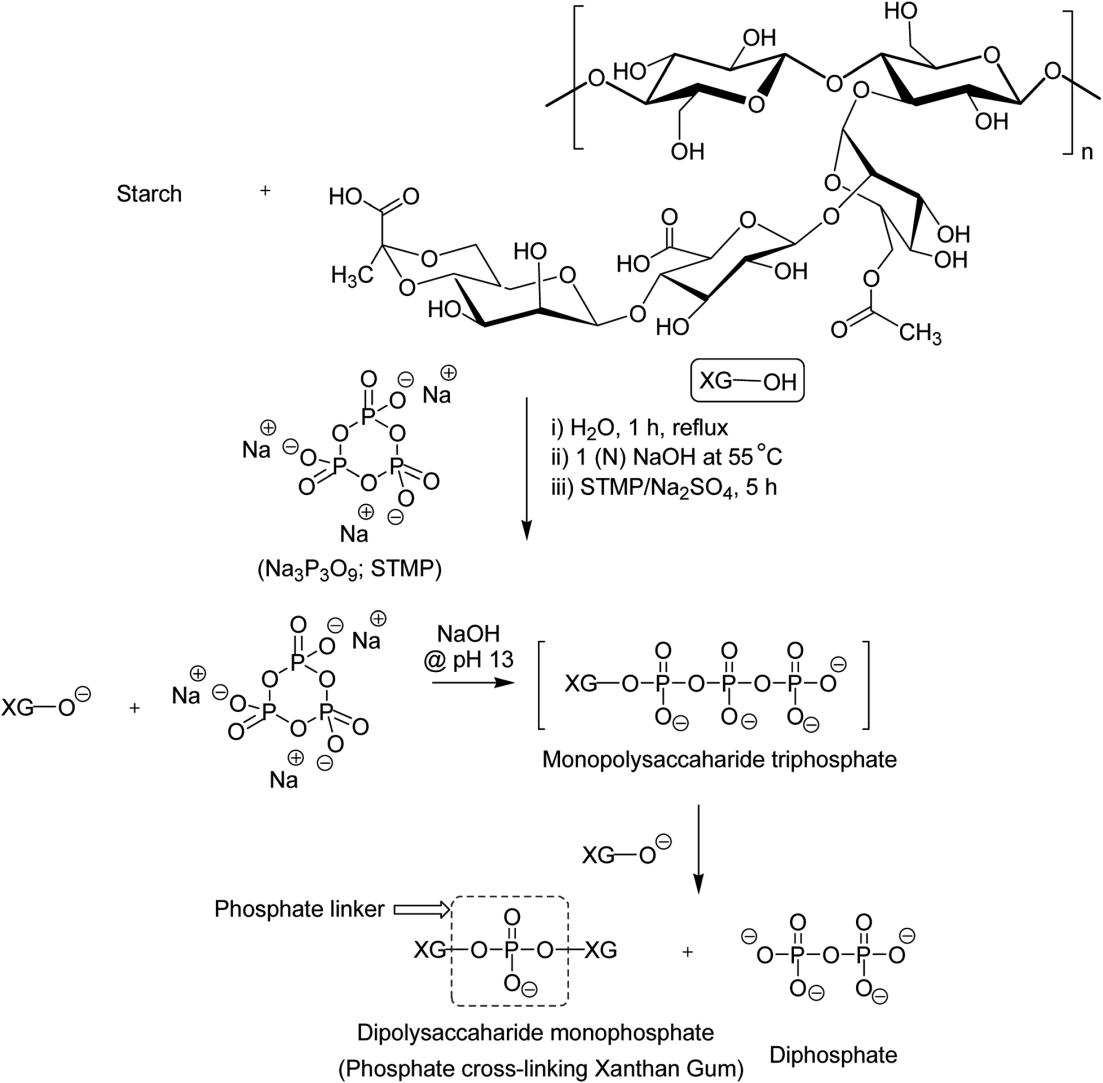
STMP-crosslinked XG derivatives.

Tao *et al.*^157^ endorsed sodium trimetaphosphate (STMP), a non-toxic and water soluble cyclic triphosphate for the crosslinking of hydroxy groups of XG chains under alkaline condition to design hydrogel disks. One phosphorous group intertwined two sugar rings of different XG chains (Fig. 6). STMP crosslinking endowed hydrogel disks more elasticity and mechanical stability than physical hydrogels. This esterification reaction induced porosity in the hydrogels, with a mean pore diameter of 114.5 ± 22.1 μm. However, the pore diameter dropped to 31.5 ± 5.5 μm at 5% (w/v) XG due to sufficient crosslinking of the matrix. XG–STMP hydrogels swelled rapidly in the first hour and attained equilibrium in PBS (pH 7.4) in 28 h. However, the hydrogel disk swelled faster in water than that in PBS and remained undissolved for more than 11 days. Regarding protein diffusion, the hydrogels recorded a slow, sustained diffusion of bovine serum albumin in PBS medium over 50 h depending upon STMP concentration. The least diffusion (∼28%) was evident at 5% STMP. STMP crosslinking perhaps introduced more anionic charges into XG, thus facilitating more water uptake by the hydrogels. Furthermore, the counter ions inside the XG–STMP hydrogels might increase the osmotic pressure and cause higher swelling.^158^

Shalviri *et al.*^158^ mingled gelatinized starch and XG in order to develop hydrogel films. STMP crosslinking produced continuous and homogeneous films with few micropores, even in absence of plasticizer. Higher STMP (20%) and XG (20%) levels speeded up the swelling of films in deionized water by 1.7-folds compared to that in phosphate buffer (pH 7.4). As usual, the gel mesh size became enlarged from 2.84 to 6.74 nm as swelling continues. The permeation of anionic drugs (ibuprofen sodium and sodium salicylate) through films was slower than neutral entities, thus demonstrating their potential for controlled release of ionizable drugs. Intermingling of a hyperbranched polysaccharide, extracted from the *Pleurotus* tuber-regium sclerotic, and XG followed by STMP-crosslinking ensued the formation of hydrogels that demonstrated shear-thinning behaviors, and improved mechanical stability. In addition, the hydrogels presented self-healing ability and controlled drug release property. The hybrid hydrogel had a storage modulus better than XG–STMP hydrogel.^159^

Bejenariu *et al.*^160,161^ highlighted the importance of STMP : XG ratio on the swelling and release of cationic dyes from the hydrogels. The hydrogels obtained at STMP : XG ratio of 10 : 1 swelled 20 times in pH 7.0 and entrapped 87.5% methylene blue. The hydrogels liberated more amounts of dyes in NaCl solution than in acid solution (pH 3.0). Overall, STMP-crosslinking supported XG hydrogels for cationic drug release.

The dehydration of hydrogel-like human nucleus pulposus and imbalanced stress distribution in the intervertebral disc causes degenerative disc disease. Thus, the regeneration of nucleus pulposus is the key to success for treating this. However, the avascularity and low mitotic activity of chondrocytes limits their successful treatment.^162^ In view of this, Leone and coworkers^163^ proposed STMP-crosslinked PVA : XG (4 : 1) hydrogels as a good substitute for nucleus pulposus.

**Fig. 7:**
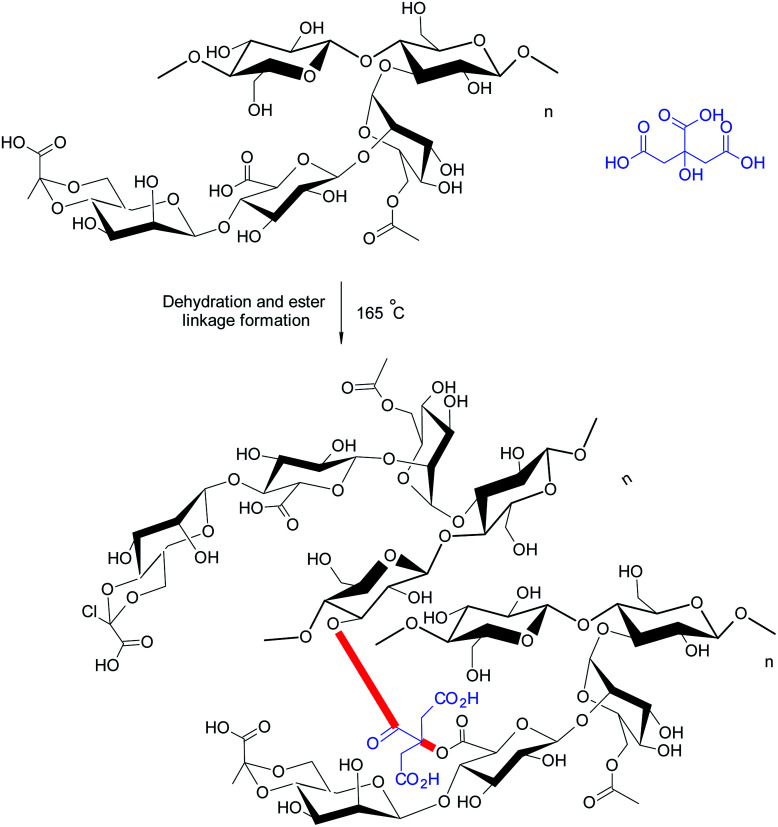
Citric acid reinforced XG derivatives.

Bueno *et al.*^21^ appraised citric acid as a natural crosslinker for the synthesis of XG hydrogel films (∼0.03 mm thick) (Fig. 7). They observed that citric acid produced porous, homogeneous films without nanofibrils, indicating its crosslinking efficiency. However, it reduced the mechanical strength of the films. The properties of citric acid-crosslinked hydrogels were comparable to those reported for xanthan–starch films.^164^ The swelling of hydrogels was significant at pH 10.0; however, the same was negligible at pH 2.0 and pH 6.5. The water absorption was initially controlled by quasi-Fickian diffusion mechanism; however switched to anomalous diffusion at later period. Perhaps, the disruption of ester bonds favored swelling of the films in basic medium. They further reported that XG chains could be intertwined by heating at 165 °C without the need of citric acid. In this case, the diffusion of water into hydrogels changed from quasi-Fickian to Fickian at later times. Heating brought about weaker crosslinking of XG chains. The nanofibrils (about 20–30 nm diameter) were evident on cryofractured hydrogel surface. Moreover, the hydrogels did not respond to pH variation promptly as was seen in case of citric acid-treated hydrogels, because they had more crosslinks to be hydrolyzed.

Circular dichroism confirmed the disordered conformation (coils) of xanthan chains in case of citric-acid crosslinked hydrogels; whereas the crosslinking by heating left XG in an ordered conformation (helixes) in the hydrogels.^165^ Under coil conformation, usually a larger number of carboxylate groups are exposed, thereby providing citric acid an opportunity to give birth to more ester linkages. The protein release behaviors from hydrogels were quite amazing. Isoelectric pH of protein and pH of release medium played a crucial role in protein release. For instance, the heat-treated hydrogels completed the delivery of BSA after 1 h over the pH range of 2–10; except at pH 4.8 close to its isoelectric point. In contrast, the release of lysozyme became very low at pH 7.0. This could be attributed to the electrostatic attraction between lysozyme and hydrogels. Both hydrogel films seemed efficient as carriers for proteins (lysozyme) having high isoelectric point. The hydrogels preserved native conformation of lysozyme after loading and thus could offer substantial bactericidal activity in wound dressings. Followed by this, Huang *et al.*^166^ designed novel wound dressing material exploiting citric acid-crosslinked transparent, porous XG films laden with silver nanoparticles (AgNPs). The nanocomposite films were non-cytotoxic to fibroblasts (L929) at <10 μg mL^−1^. The dressing prohibited the formation of biofilms, reduced the inflammatory reactions, and triggered angiogenesis of the granulation tissues in non-healing wounds, infected with methicillin-resistant *S. aureus*. The nanocomposites impeded immature release of AgNP in PBS solution and hence exhibited the antibacterial effect for more than 24 h.

**Fig. 8:**
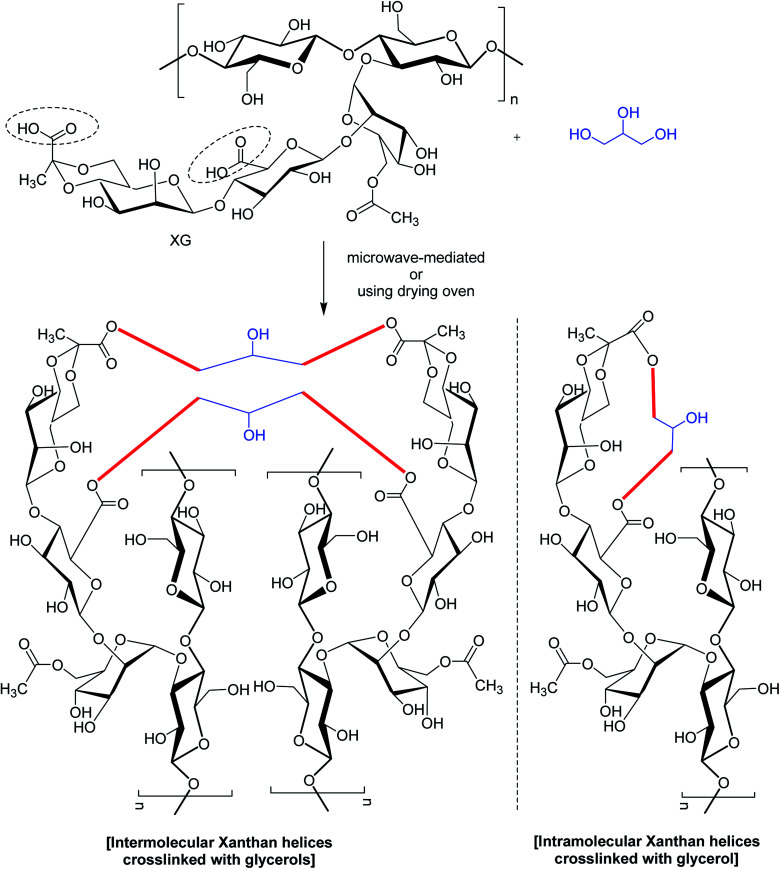
Glycerol-crosslinked XG.

In a study,^167^ glycerol was mentioned for chemical bonding of XG in a waterless or near-waterless environment at temperature >105 °C (Fig. 8). It was hypothesized that water generated during cross-linking reaction could catalyze the unwinding of double-helix of XG and provide more access to its functional groups and backbone for cross-linking. The crosslinking occurred at glycerol : xanthan ratio of <27.6. Higher xanthan : glycerol ratio imparted more hardness, swellability (>39 g water per g gel), dye sorptivity (>85%) to the hydrogels. The hardness was almost 40 times greater with 50% xanthan gel than that made with 5%. The interdiction of red 40 and blue 1 (food dyes) release in water (60–70% in 24 h) unfolded the possibilities for constructing slow-release matrices. Moreover, the crosslinked gels came up effectively with dye absorbent capacity; thus advocated its application in food, pharmaceutical and other industries.

Hamcerencu *et al.*^168^ described esterification of XG with an unsaturated acrylic acid or acid reactive derivatives (acryloyl chloride, maleic anhydride) followed by grafting with *N*-isopropylacrylamide. This resulted in thermo- and pH-sensitive hydrogels. Maleic anhydride presented higher reactivity towards primary hydroxy groups (C6) as compared to others. Higher temperature and reaction time had positive impact on the degree of substitution.

**Fig. 9:**
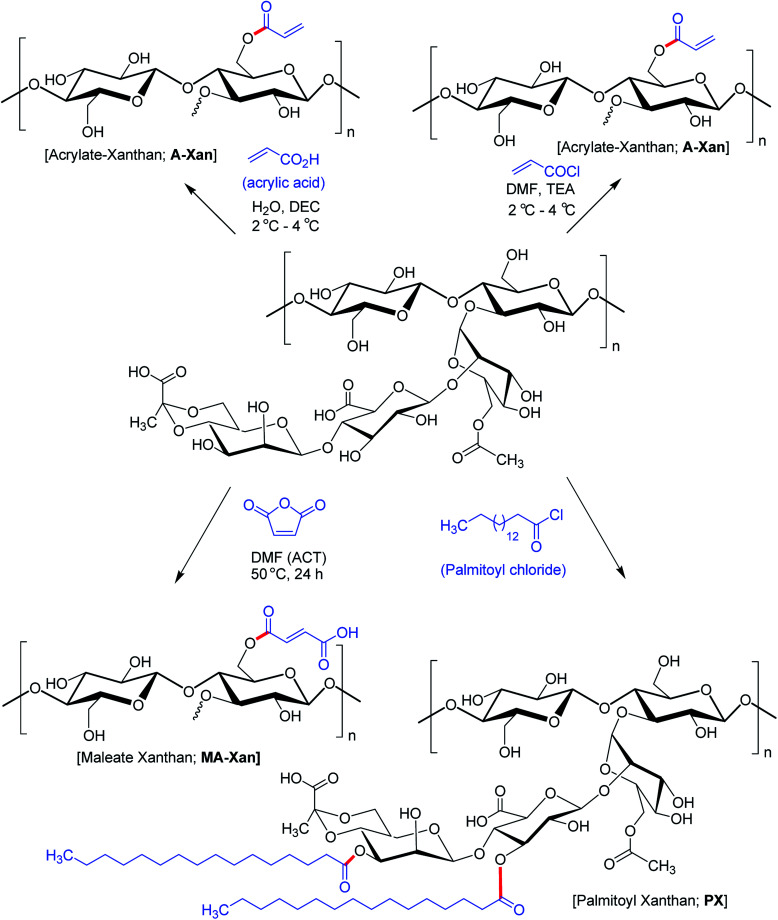
Chemical structures of acrylate XG, maleate XG and palmitoyl XG.

Mendes *et al.*^169^ evinced that palmitoylation of xanthan (xanthan : palmitoyl chloride = 1.7) expedited self-assembly of XG and furnished a compatible environment for cell microencapsulation. However, there was a dearth of physical stability to enclose cells and protect from immunotoxins. Subsequent membrane coating of the microcapsules with poly-l-lysine allowed permeation of sufficient oxygen and nutrients necessary for cell survival and proliferation (Fig. 9).

**Fig. 10:**
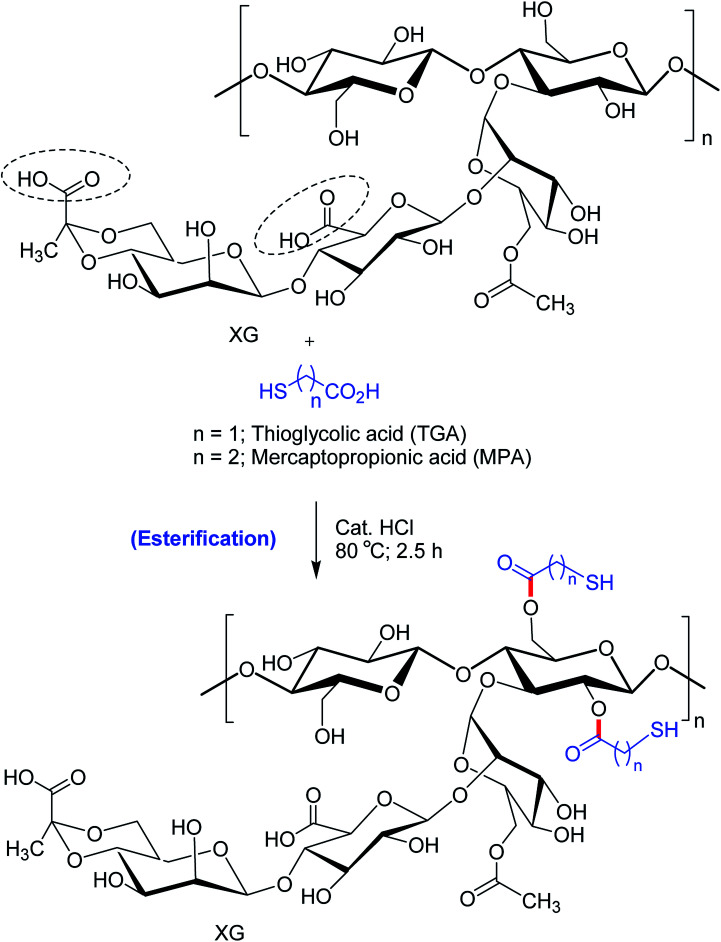
TGA- and MPA-derived modified XG.

Bhatia *et al.*^170^ pointed out that thio-esterification of XG with mercaptopropionic acid and thioglycolic acid evoked better *ex vivo* mucoadhesion (Fig. 10). The extent of mucosal retention of metronidazole-loaded buccal pellets of XG–mercaptopropionic acid was less than XG–thioglycolic acid. Improved mucoadhesion of thiolated XG over native XG could be credited to the disulfide links between mucus and thiol groups. Nonetheless, the buccal pellets of XG–thioglycolic acid sustained the diffusion of metronidazole more effectively than XG–mercaptopropionic acid and native XG pellets. The degree of thiol substitution in XG–thioglycolic acid was 1.07 times higher than XG–mercaptopropionic acid. This could be responsible for variable *ex vivo* bioadhesion and *in vitro* release characteristics of the two conjugates.

In view of the potential toxicity of surfactants as suspending agents, Skender and groups^171^ synthesized three ester derivatives of XG using diphenylmaleic anhydride, phthalic anhydride, and epichlorhydrin–phenol and assessed the worth of these derivatives as dispersing agent for double-wall carbon nanotubes in water. Xanthan itself is a very poor dispersing agent for carbon nanotubes. However, these derivatives proved useful as stabilizer for suspension of carbon nanotubes (0.5%, w/w). Xanthan esterified with diphenylmaleic-and phthalic-esters successfully immobilized carbon nanotubes in very acidic media compared to that esterified with epichlorhydrin–phenol. Above pH 5.0, all the derivatives stabilized the suspensions effectively. Summarily, the presence of aromatic moiety in XG backbone was essential in dispersing carbon nanotubes in water *via* p–p stacking interaction.

A group of investigators synthesized succinoyl XG following reaction with succinic anhydride in presence of 4-dimethylaminopyridine catalyst at room temperature.^172^ Succinoyl XG turned into unflowable hydrogels at a very low strength (1.4% w/w), while the unmodified XG existed as flowable fluid at the same concentration.

In case of native XG, the hydrogel network is usually formed through physical entanglements. Succinoylation promoted the formation of secondary bonds, rendering more elasticity to the hydrogels. Further, the hydrogels responded to ionic strength and sustained the release of gentamicin for 9 days in PBS (pH 7.4). The hydrogels inhibited the formation of biofilm and demonstrated excellent bactericidal function in rabbit subcutaneous *S. aureus* infection model. As was evident from cytocompatibility study against human lens epithelial cells, the hydrogels supported cell adhesion; proliferation and migration at a dose of 1 mg g^−1^. Overall succinoylated XG hydrogels were propitious as drug delivery materials for antibacterial applications.

### 2.3. Oxidized XG

Oxidation of XG could partially convert glucopyranose units to aldehyde and create additional functional sites for covalent crosslinking. As reported with alginate polysaccharide, the sodium periodate oxidized the hydroxy groups on C2 and C3 of the repetitive unit of alginate and led to the formation of two aldehyde groups in each oxidized monomeric unit by the rupture of C2–C3 bond.^173^

Guo and coworkers^174^ brought into play xanthan aldehyde as a crosslinker for producing transparent, UV-protective edible gelatin films. Increased C=N groups by Schiff's base formation greatly improved the hydrophobicity, mechanical properties and thermal stability of gelatin films. Paiva and colleagues^175^ exploited XG aldehyde as an adhesive for the natural cork stopper. The authors proclaimed that oxidized XG performed well due to the reaction of aldehyde groups with hydroxy groups on cork surface during high temperature drying. Ma *et al.*^176^ disclosed a new hydrogel system based on XG aldehyde and phosphatidylethanolamine liposome. The hydrogel was sensitive to stimuli like heat, pH, and histidine and papain enzyme. The hydrogels manifested self-healing ability and provided an excellent environment for cell encapsulation for tissue engineering.

Intrinsic healing property of hydrogels is of utmost importance for *in vivo* drug delivery because this property could relieve hydrogels from destabilization under adverse mechanical or chemical exposure *in vivo*.^177^ Salazar and associates^178^ reported intrinsic self-healing ability of a composite hydrogel system of oxidized XG and chitosan. The hydrogels extended successful self-healing ability at room temperature and pressure while maintaining a good mechanical strength at equal chitosan : aldehyde XG mass ratio.

**Fig. 11:**
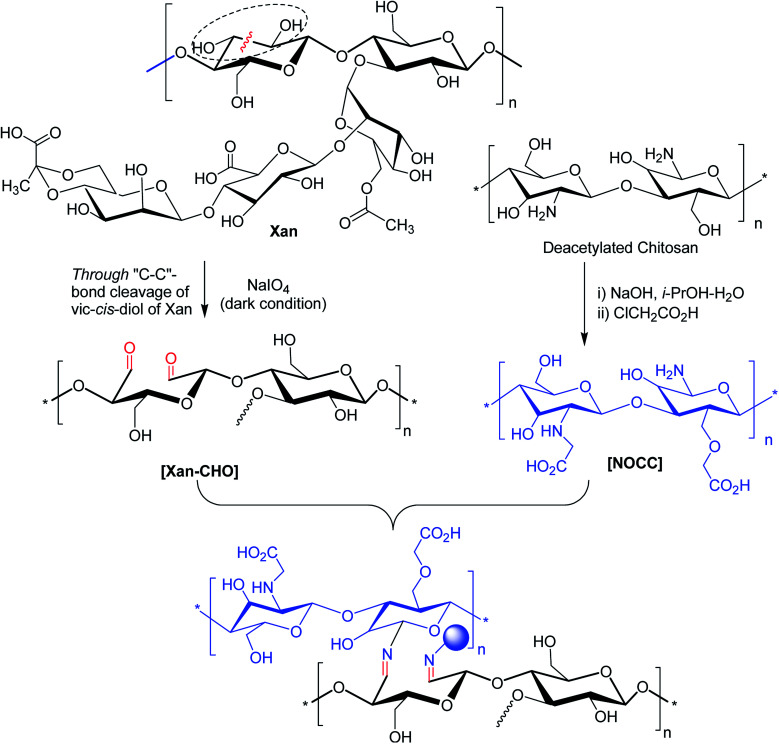
Formation of XG–CHO/NOCC hydrogel *via* imine formation.

Injectable hydrogels have been abortive due to their instability to tissue exudates and delayed gelation under physiological conditions. Huang *et al.*^179^ attempted to overcome these issues. They devised a stable and rapid gelation method for fabricating self-healing hydrogels based on hydrogen-bonding interaction and Schiffs base reaction between XG aldehyde and carboxymethyl chitosan (Fig. 11). The incorporation of vascular endothelial growth factor (VEGF) in hydrogels speeded up the reconstruction of abdominal wall in rats. The hydrogel managed the release of protein effectively.

The antioxidant activity of oxidized XG was also disclosed in the literature. Xiong *et al.*^180^ exposed that XG when oxidized under alkaline medium exhibited better antioxidant activity than native XG. XG oxidized under acidic condition but having similar molecular weight had relatively poor antioxidant activity. The variable contents of pyruvate and reducing sugar could be responsible for this discrepancy. Nevertheless, dialdehyde XG could serve as a reducing agent for the synthesis of silver nanoparticles as well as designing biocompatible composite dressings suitable for burn healing.^181^

### 2.4. Amide functionalized XG

**Fig. 12:**
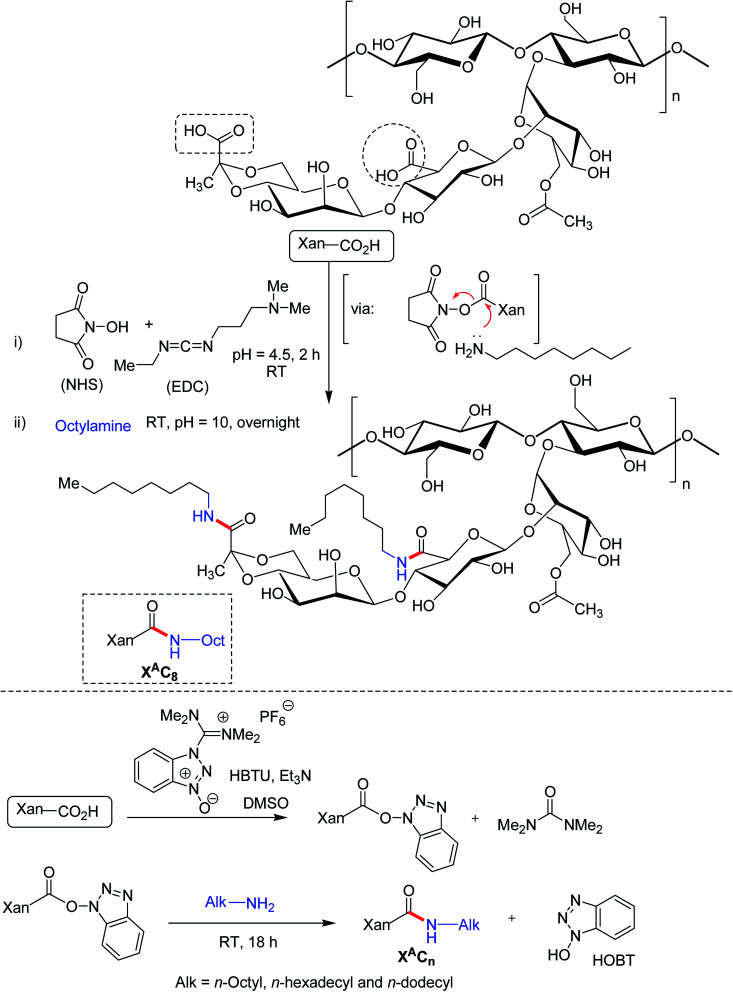
Long alkyl chain conjugation to XG under its ordered and disordered conformation.

The carboxy functionalities of XG chains could be the focal points for bifunctional amine crosslinkers to establish covalent amide bonds. Long hydrophobic alkyl chain can be substituted to XG backbone under ordered (helical) and disordered (coil) conformation *via* carbodimiide chemistry. Roy *et al.*^182^ conjugated octylamine onto the carboxy moieties of xanthan under its ordered (helical) conformation in water at room temperature (Fig. 12). The reaction involved complete protonation of the carboxy functions at pH 3.0 followed by its activation in presence of EDAC/NHS. The pH was adjusted afterward to 4.5 which promoted the protonation of EDAC's nitrogen atoms. Subsequent addition of an aqueous solution of octylamine, pH adjustment (pH 10.0) led to the formation of amide bonds through the nucleophilic attack of octylamine onto the activated carboxy functions of xanthan. The grafting density varied between 8 and 29% depending upon the stoichiometry of octylamine and EDAC with respect to carboxy functions. In spite of additional intermolecular interactions, hydrophobic chain grafting did not alter the viscoelasticity, and chain conformation of XG. Even being amphiphilic, the modified XG did not exhibit any additional self-assembling properties. Perhaps, the high stiffness of xanthan helices precluded self-assembly of modified XG, usually observed for flexible amphiphilic polymers. However, the hydrophobic interactions strengthened the suspending ability of xanthan considerably at rest but its shear-thinning behavior remained untouched at high shear rate. This property could be tuned by changing the grafting density. Thus, ordered modification of XG could enrich the list of potential stabilizers and thickeners in pharmaceutical formulation. As observed earlier by Roy *et al.*,^182^ the modification of XG under ordered conformation exhibited similar behavior to native XG; albeit the chain relaxation occurred slowly. Conversely, XG when modified under its disordered conformation displayed a chemical gel-like behavior without any relaxation of the chain.^183^ The gel formation took place as low as 0.05 g L^−1^ concentration. This owed to the presence of flexible backbone and squealed to intermolecular hydrophobic interactions. Circular dichroism study unveiled the fact that acidic form of xanthan adopted disordered conformation in DMSO. Further, they altered the structure of XG under its disordered conformation using *O*-(benzotriazol-1-yl)-*N*,*N*,*N*′,*N*′-tetramethyluronium hexafluorophosphate (HBTU) as reagent for acid-amine coupling. Briefly, acidified XG was solubilized into DMSO at 80 °C and was then cooled to room temperature. Then, HBTU, triethylamine (deprotonating agent for carboxylic acid) and octylamine were introduced. Fantou *et al.*^184^ synthesized octyl derivative of XG under its ordered conformation. The octyl XG had an exceptional capacity to stabilize oil-in-water emulsion at 0.2% w/w concentration, without the need of additional surfactant. The grafting density (8–32%) had negligible effect on emulsion stability. This finding opened up the possibilities of formulating emulsion that requires little or no surfactant in order to limit their toxicological and environmental impact.

However, the study findings on emulsion containing octyl xanthan (disorder conformation) thwarted emulsion stability through creaming, regardless of its concentration. The instability problem became more pronounced at higher grafting density. However, this phenomenon reversed for *n*-hexadecylamine XG (disorder conformation).^185^

**Fig. 13:**
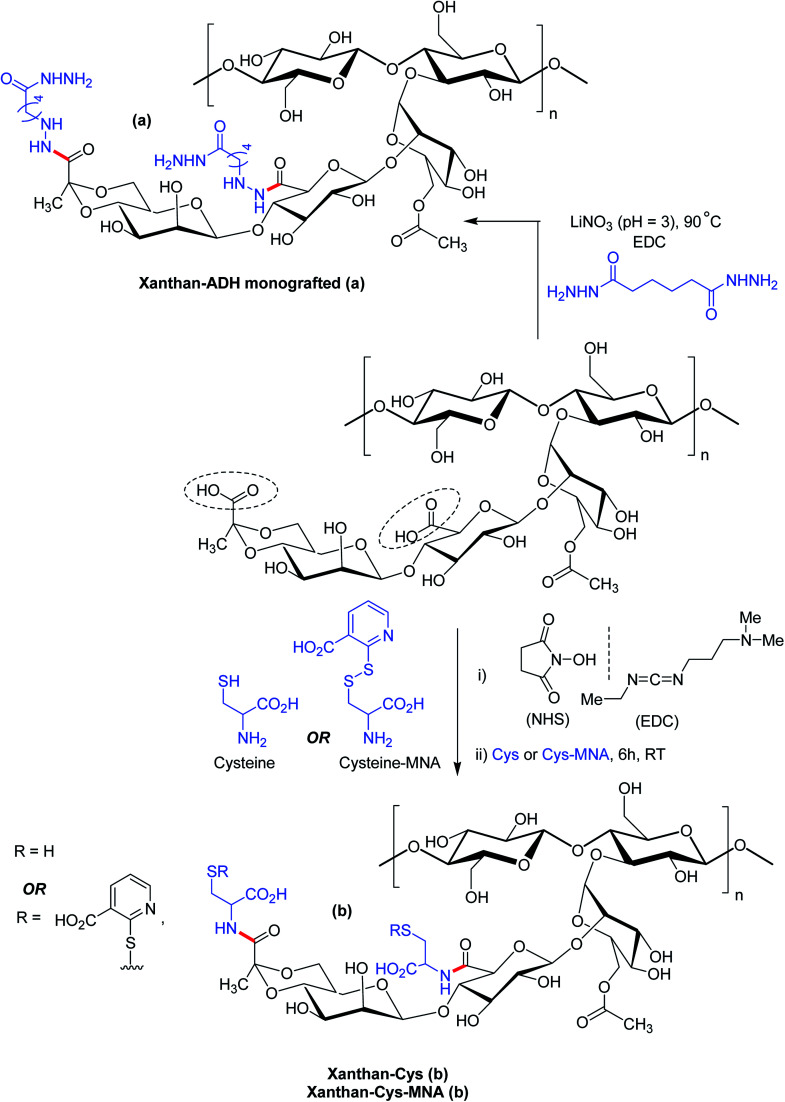
Synthesis of (a) ADH-reinforced XG and (b) XG-cysteine or XG-cysteine–MNA.

Bejenariu *et al.*^161,186^ reported a new XG hydrogels based on adipic acid dihydrazide (ADH) treatment. The crosslinking of XG with ADH is displayed in Fig. 13a. In the crosslinking reaction, LiNO_3_ was used for the stabilization of the helicoidal structure of XG at pH 3.0. The hydrogels exhibited pH-dependent swelling but the pH-effect was not so drastic. The least degree of swelling was noted at pH 3.0 because the helical conformation of XG predominated in crosslinked networks. Despite the prevalence of coil conformation, the weak amide bonds restricted the networks from exhibiting similar swelling behaviors at pH 13.0 to that noted under acidic condition. Notably, the highest degree of swelling was recorded at neutral pH. The hydrogels absorbed ∼98% of methylene blue in 24 h and released the dye in salt solution at a rate faster than in acidic solution (pH 1.5). Laffleur and groups^187^ showed that thiolated XG disc worked better in terms of stability (1.62-fold), *ex vivo* buccal mucoadhesion (8.35-fold) and tensile strength (2.65-fold) compared to unmodified xanthan. These findings coupled with its non-cytotoxicity towards Caco-2 cells inspired the authors in developing buccal patch to treat sialorrhea, a buccal disease associated with an involuntary loss of saliva. To fight against enhanced psychosocial and mental problems of sialorrhea patients,^188^ tannic acid was found suitable for inhibition of excessive flow of saliva. Laffleur *et al.*^189^ developed l-cysteine-conjugated XG buccal patch for tannic acid. The constant swelling in simulated saliva solution (pH 6.75) and non-cytotoxicity to Carey 24 cell lines embellished the buccal patch. Thiolation prohibited matrix erosion about five times and lessened the saliva flow about 33% more than the unmodified XG in excised porcine buccal mucosa. Mucoadhesive and water vapor uptake properties of XG became more pronounced after thiolation of XG. The patch released tannic acid half as fast as XG in 6 h in simulated saliva solution and thus opened an avenue for sialorrhea research. Menzel *et al.*^190^ adopted a distinct strategy for thiolation of XG. The process comprised of coupling reaction of 2-mercaptonicotinic acid (MNA) to l-cysteine by disulfide exchange and direct attachment to XG *via* carbodiimide mediated amide bond formation (Fig. 13b). XG-cysteine–MNA concurred about 1.7-fold and 2.5-fold higher mucoadhesion than cysteine-XG (XG-Cys) and XG *ex vivo*, respectively. In the presence of H_2_O_2_ oxidizing agent, XG-cysteine–MNA showed high stability towards oxidation. Reversible ciliary beat frequency in porcine nasal epithelial cell culture indicated low toxicity of all xanthan derivatives. The *in situ* gelling behavior of XG-Cys–MNA was independent of polyvalent cations, thus hinted safe application of aqueous solutions.

**Fig. 14:**
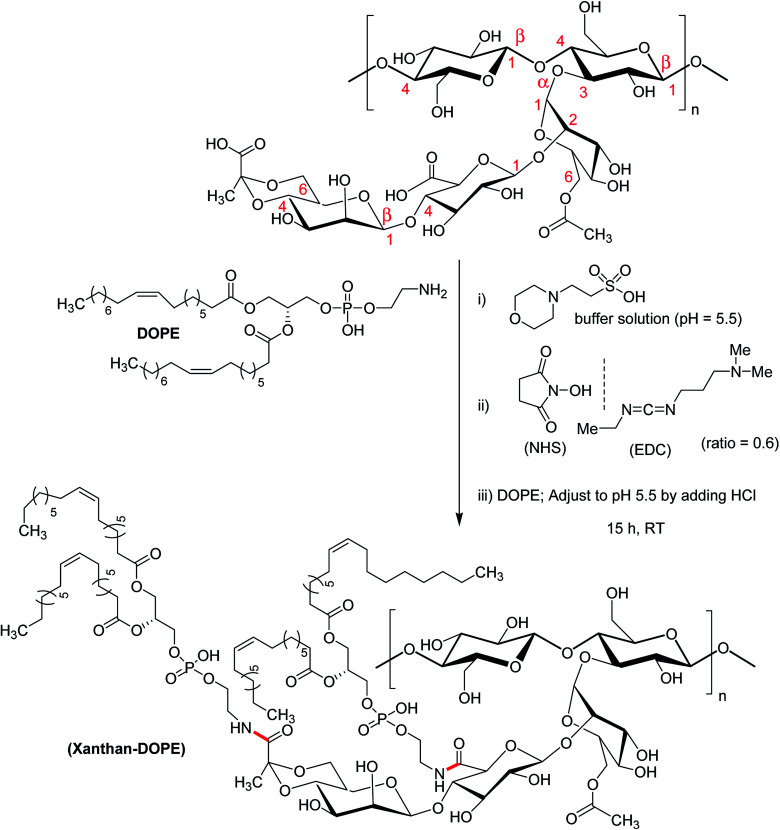
Synthesis of amphiphilic DOPE–XG.

Mendes *et al.*^191^ synthesized an amphiphilic XG following conjugation of phospholipid (1,2-dioleoyl-*sn*-glycerophosphoetilamine, DOPE) to the anionic XG (Fig. 14). Considering two carboxy groups in xanthan repeating unit, the degree of substitution was estimated to about 1.16 ± 0.024. The investigators applied microfluidic approach for the production of microcapsules for cell entrapment. Coating with 0.1% poly (l-lysine) improved the mechanical strength of the capsules. The viability of ATDC5 cells remained unaltered after encapsulation. The droplets generated through microfluidic approach and the self-assembly of xanthan–DOPE produced microcapsules with an environment compatible for cell survival and proliferation.

The antimicrobial activity of lysozyme is limited to Gram positive bacteria, but its antibacterial spectrum can be extended towards Gram negative bacteria after carbohydrate conjugation through the Maillard reaction. Hashemi *et al.*^192^ synthesized xanthan gum-lysozyme (1 : 1) conjugate under mild Maillard reaction condition (sodium phosphate buffer, pH 8.5 and 60 °C for 10 days). It was found that approximately 1.9 mmol XG could be attached to one mole lysozyme. Lysozyme–XG conjugates showed pH- and temperature-dependent solubility, and heat stability with improved emulsion and foaming properties. The lysozyme conjugation significantly impeded the growth of *Staphylococcus aureus* and *Escherichia coli* in a dose dependent manner. These findings broaden the application of this enzyme in food or pharmaceuticals.

### 2.5. Acetalated XG

**Fig. 15:**
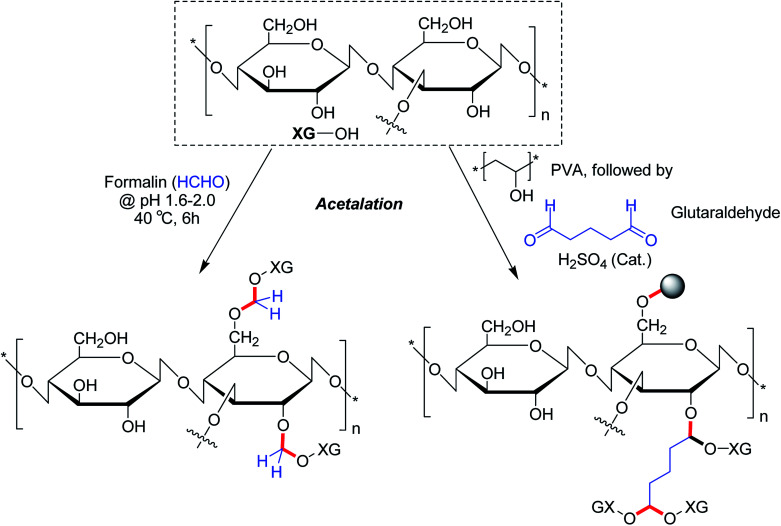
Acetalation of XG using formaldehyde and glutaraldehyde.

The alcoholic hydroxy groups could react to aldehydes under acidic condition. Thus, the properties of XG could be modified through the formation of acetal linkages in presence of aldehydes. Su *et al.*^104^ proclaimed that formaldehyde treatment gave birth to acetal linkages in XG. Despite a loss in crystallinity, the acetalation reaction caused enhancement of viscosity and solubility of XG. Ray *et al.*^193^ described a microparticulate system where they used glutaraldehyde for acetalation of XG and PVA. Regardless of gum : PVA ratio and crosslinker concentration, the microparticles showed 84% drug entrapment efficiency. The crosslinker concentration declined the rate of delivery of entrapped diclofenac molecules considerably *in vitro*. On contrary, XG promoted the drug release rate in biological fluids. Preclinical trials demonstrated higher relative bioavailability (1.69 times) having a good *in vitro-in vivo* correlation. The pioneering work of Ray *et al.*^193^ enlightened us on the potential utility of glutaraldehyde as a crosslinker for XG for controlled drug delivery applications. However, the toxicity profile of glutaraldehyde poses a major concern which perhaps precludes its use as chemical crosslinker for drug delivery matrices (Fig. 15).

### 2.6. Physically modified XG

XG could be physically modified using various treatments like heat, moisture, annealing, dry heating to change its physicochemical properties. Sereno *et al.*^194^ extruded XG under water flow in a twin screw extruder with co-rotating screws. It was then dried in a vacuum oven at 65 °C for 72 h under a pressure of 100 Pa to retain final water to <8%. The viscosity and dispersibility of 0.75% XG in distilled water was appreciably improved after extrusion processing. Foster and Mitchell^195^ noted that the extruded XG behaved like polyelectrolyte particles, demonstrating excellent dispersibility and a strong salt dependence on the degree of swelling. Heating of the extruded XG dispersion resulted in viscosity development identical to starch, citing end use advantages for XG. In another study,^196^ it was found that high pressure homogenization (HPH) (276 MPa) precipitated viscosity of XG solution, possibly due to disruption of structural network.^197^ Further, this mechanical treatment reduced molecular weight and caused an increase in the hydrodynamic volume and polydispersity of the polymer remarkably. The treatment further led to irreversible change in the rheological and structural characteristics of XG. The HPH-modified XG responded differently to temperature, compared to that observed with XG after low pressure homogenization (LPH, 69 MPa). LPH caused sharp lowering of storage modulus (elasticity) around 50 °C; whereas HPH-treated XG exhibited similar trend around 40 °C. Zhang *et al.*^198^ elucidated the swelling, mechanical, rheological and adsorption properties of PVA–XG hydrogel synthesized over three freezing (−15 °C for 24 h) and thawing (room temperature for 1 h) cycles. They postulated that during freezing and thawing of PVA–XG solution, the hydroxy and carboxy functional groups of the polymers assumed a three-dimensional network through hydrogen bonding interactions, and formed a physical hydrogel. The wrinkles were evident on the hydrogel surface, especially at high XG concentration. Increase in specific surface area of the network, in turn made the hydrogel more elastic in terms of absorption or release of water, anions or cations through a large number of contact points established between the hydrophilic chain and the solvent. A distinct fluid characteristic was noticed. At low shear rate, hydrogels behaved like a Newtonian fluid; whereas the same turned into pseudoplastic non-Newtonian fluid at high shear rate. The shear stress of the composite hydrogel increased with increasing concentration of XG and reached maxima at PVA : XG ratio of 12 : 8. Additionally, the apparent viscosity and shear thinning characteristics of XG progressively increased at higher strength. At higher XG content, more hydrophilic groups entangled into regular network structure by hydrogen bonding and van der Waals forces, which enabled wrapping of more free water. Consequently, the viscosity of the composite solution became larger and pseudoplasticity came into existence.^198^ The PVA–XG (12 : 8) hydrogels swelled abruptly at high pH and attained a maximum equilibrium swelling index of 1.3 at pH 8.0. This indicated that alkaline pH favored the adsorption of methylene blue by composite hydrogels. XG contributed to the improved swelling of PVA–XG hydrogels; however reduced the compression properties owing to the formation of hydrogen bonds between polymers. Ultimately, the mechanical properties of the hydrogel diminished. The maximum adsorption of methylene blue dye was noted at PVA : XG ratio of 12 : 8. A strong electrostatic attraction between XG and dye could contribute to this effect. The recycling ability of hydrogels strongly supported its application in dye removal from waste water. Shimizu *et al.*^199^ observed that the addition of 0.2% XG in 17% PVA solution minimized liquid viscosity while maintaining mechanical properties and pourability into injection molds during the fabrication of 3D models. The addition of XG facilitated the fabrication of biomodels through gelation of PVA/XG solution in water : DMSO (20 : 80) at −30 °C for 24 h. Bhunia and coworkers^200^ prepared PVA (10%) : XG (1–5% w/w) hydrogel films following irradiation at 5 kGy dose under high energy electron beam and investigated the influence of low molecular weight (1.4 kDa) PVA (LPVA) and high molecular weight (11.5 kDa) PVA (HPVA) on the physical and mechanical properties of the film. The physical properties of the films under dry state remained indistinguishable but HPVA–XG hybrids were superior in terms of wet-mechanical properties and diltiazem release. Tensile strength of HPVA–XG films was excellent at 1% XG and the same was much less in LPVA–XG films. The degree of swelling became intensified with increasing XG content in HPVA hydrogels.

In LPVA–XG hydrogels, the swelling dropped drastically on addition of 1% XG, but eventually increased at 3% and 5% XG concentration. Thus, the most physico-mechanically balanced hydrogel membranes were obtained at 1% and 5% XG for HPVA and LPVA, respectively. A sharp loss in crystallinity in presence of 1% XG indicated huge loss of cohesive interaction and establishment of higher HPVA–XG interaction. On contrary, gradual increase in XG content up to 5% produced greater crystallinity in LPVA hydrogels. LPVA was surprisingly more viscous than HPVA^201^ and hence, LPVA–XG produced better phase compatibility, particularly at higher XG content. On contrary, intermingling of HPVA and XG generated better phase mixing at low XG content but led to huge phase separation at elevated XG level due to viscosity mismatches. LPVA showed better phase mixing with 5% XG. In HPVA : XG (1%) films, the diltiazem molecules were not uniformly distributed because of their different phase viscosity, thus failed to retain drug appreciably alike LPVA : XG (5%) films. The drug–PVA–XG hydrogen bonding interaction dictated the entrapment of diltiazem within PVA–XG hybrid. High flexibility in LPVA : XG (5%) thus allowed more diltiazem to elute in swelled state despite their better drug entrapment efficiency than HPVA : XG (1%) films. Regardless of XG concentration, the drug release was insignificant (2.5–4.6% in 12 h). The investigators achieved success in suppressing the burst release of drug from PVA hydrogels following exposure to irradiation. Disha *et al.*^202^ prepared hydrogels by heating XG, sodium benzoate, and potassium sorbate and glycerin mixture at 85 °C. The biodegradability characteristics of XG persisted over the concentration range of 2–3%. The hydrogels were devoid of antimicrobial activity against *E. coli* and *Klebsiella* spp. The physical crosslinking (H-bonding interactions) of XG with poly (*N*-vinyl imidazole) enabled Sabaa and coworkers^203^ to fabricate hydrogels containing bovine serum albumin (BSA). Depending on variation in gelation time, BSA loading and polymer concentration, the protein entrapment efficiency escalated to 99.17%. Higher polymer concentration promoted BSA release in PBS. The biocompatibility of hydrogel was ascertained on VERO cell line. SDS-PAGE analysis confirmed the structural integrity of protein after encapsulation or release.

Intra-articular injection of hyaluronic acid could be effective in treating osteoarthritis due to its lubricating and cushioning properties.^204,205^ However, hyaluronic acid rapidly degrades *in vivo* by hydrolytic or enzymatic reactions.^206^ Henceforth, a polymer which is similar to hyaluronic acid in structure and function, but with a longer effect in the joint is needed. XG is similar to hyaluronic acid in rheology and viscosity.^207^ Additionally, XG solution is very stable in a wide range of pH, ionic strength and temperature.^208^ XGlyase is mainly produced by bacterium;^209^ therefore XG is not easily degraded *in vivo*. Intra-articular injection of XG could be an effective therapy for osteoarthritis. Han *et al.*^210^ extensively purified XG, dissolved in buffered physiological saline and sterilized at 121 °C for 15 min and evaluated the protective effect of XG injection on articular cartilage. Results indicated that injection of XG could protect the joint cartilage and reduce papain-induced osteoarthrotis progression in rabbit model. The same result was obtained at an injection frequency lower than HA. This finding seemed promising in the development of a new therapeutic method for osteoarthritis. Bueno *et al.*^65^ synthesized XG–hydroxyapatite particles following homogenization of XG solution with Ca(OH)_2_ and H_3_PO_4_ solution under pH 7.5. Similarly, xanthan strontium-substituted hydroxyapatite particles were obtained using Ca(OH)_2_, Sr(CH_3_COO)_2_, xanthan solution and H_3_PO_4_ solution at pH 7.5. Initially Ca^2+^ ions were dissolved at pH 10.0 in presence of xanthan chains. Calcium ions were chelated by xanthan chains, forming nucleation sites for hydroxyapatite crystal growth. As phosphoric acid was added to the solution, the medium pH decreased to ∼7.5, promoting the precipitation of Ca^2+^ ions as hydroxyapatite. XG–hydroxyapatite and xanthan strontium-substituted hydroxyapatite nanoparticles presented mean size of 56 ± 18 nm and 75 ± 18 nm, respectively. Xanthan aqueous solution containing nanoparticles was casted into xanthan-based nanocomposite hydrogels in presence of citric acid. The dehydration crosslinking was accomplished by heating the films at 165 °C for 7 min. The nanocomposite films were apparently homogeneous. Hence, the presence of xanthan chains on the surface of particles provided an indication of compatibility between particles and xanthan chains. Nanocomposites presented porous structure and the incorporation of Sr or xanthan–hydroxyapatites did not affect the surface energy and consequently, posed no substantial influence on osteoblast proliferation. On the other hand, the chemical composition of nanocomposites influenced ALP activity. ALP is a cell membrane glycoprotein that catalyzes the hydrolysis of phosphate esters in alkaline pH and thus plays an important role in the mineralization process of bone matrix. Its activity in human serum is used as indication for diseases related to liver and/or bone. It was noted that the osteoblast ALP activity increased as the filler content was increased from 10% to 30% in both the nanocomposites, but this enhancement was much more pronounced in case of citric acid crosslinked XG–strontium hydroyapatite. In fact, the presence of strontium in hydroxyapatite structure supported osteoblast and osteoclast growth. Nanocomposites made of crosslinked xanthan chains and xanthan hydroxyapatite (10%) was homogeneous and mechanically stronger than bare xanthan networks or nanocomposites with hydroxyapatites. Mixing of ZnO nanoparticles (size 15–25 nm) with XG–PVA mixture followed by irradiation at 30 kGy led to the generation of biocompatible XG–PVA/ZnO nanocomposite dressing hydrogels.^211^ Homogeneous porous hydrogel network and the presence of ZnO nanoparticles provided an excellent fluid uptake capacity in pseudo-extracellular fluid (554–664%) and water (1281–1603%). The water retention capacity (about 50–65% after air exposure for 6 h) and water vapor transmission rate (167–184 g m^−2^ h^−1^) was sufficient to keep wound's surface moist. ZnO dressings exhibited profound antimicrobial activity against *Staphylococcus aureus*, *Escherichia coli* and *Candida albicans*. To avoid lumps and accelerate the dissolution of XG in water, Zhao *et al.*^212^ modified the surfaces of XG particles in dimethylbenzene with organometallic complex COMe at pH 4.5 adjusted by citric acid. The modification didn't change the molecular structure of XG; however weakened the hydrogen bonding interaction between XG molecules. Dispersible XG powders were completely dissolved in simulated seawater in 35 min without lumps. It was reported that sequential control of wetting and drying of XG produced sticky samples, thus posed difficulty in handling.^213^ Co-grounding of this treated XG with mannitol (1 : 1) improved the flowability and crystallinity marginally. Co-grounding afforded excellent water loading capacity (60.93%) and thus appeared to be an effective disintegrant for roxithromycin orodispersible tablets. The modified XG (10% w/w) and microcrystalline cellulose (MCC) (15%) allowed the disintegration of tablets in less than 60 s. At 1 : 3 ratio of co-grounded XG : microcrystalline cellulose, the tablets even disintegrated in about 14 s. The tablets reduced the lag time 9-fold and remained stable over a period of 12 months. Highly porous structure of modified XG and higher level of MCC promoted faster wetting (6.52 ± 0.42 s) and water absorption (150%). A marginal difference in drug release was noted at salivary pH (pH 6.4) and physiological pH (pH 7.4) (∼91–93% in 30 min). Higher percentage of drug release could provoke drug absorption through buccal cavity. Above all, the modified XG behaved like a directly compressible excipient and exhibited swelling dynamics suitable for use in rapidly disintegrating tablets.

## 3. Conclusion

This review inferred that the physicochemical and mechanical properties of virgin XG could be tailored *via* etherification, esterification, oxidation, acetalation and amidation for specific end use. The ionic and/covalent crosslinking of XG allowed the fabrication of hydrogel films, hydrogel beads, matrix tablets for oral and transdermal drug delivery applications. The conjugation of long alkyl chain to XG backbone triggered self-assembly of XG and facilitated incorporation of substantial amount of drug molecules in the lipophilic confines of nanomicelles. The octyl XG unveiled its potential use as emulsifying agent. l-Cysteine conjugation to XG further assisted in developing buccal patch for sialorrhea treatment. Even, the phospholipid conjugation to anionic XG followed by poly (l-lysine) crosslinking proffered an environment adequate for cell survival and proliferation. Thiolation markedly improved mucoadhesion of XG. The esterification of XG further conferred self-healing as well as sustained drug release properties. The esterified hydrogels seemed suitable as a substitute for human nucleus pulposus. The self-healing feature of aldehyde XG–carboxymethyl chitosan hydrogels was also found promising.

The acetalated XG hydrogels improved the pharmacokinetics of entrapped drug remarkably. The physical treatments such as extrusion, freezing–thawing and high shear homogenization greatly improved the mechanical properties of XG. In addition, the physically crosslinked XG hydrogels had immense potential in encapsulating proteins without alteration of its structural integrity. The intra-articular injection of purified XG on articular cartilage was found effective as well against osteoarthritis. The XG-based nanocomposites had shown excellent mechanical stability, osteoblast ALP activity as well as wound dressing capability. Even, simple co-grounding of XG with mannitol caused reasonable improvement in flow characteristics of XG. Overall, the findings suggested that the functionalized XG had great potential for pharmaceutical and biomedical applications. However, the *in vivo* potential of these novel XG derivatives are yet to be assessed explicitly in order to unveil their safety and efficacy towards drug delivery and biomedical applications. This review would certainly encourage the pharmaceutical and biomedical researchers to explore other polysaccharides for developing newer materials for commercial use.

## Conflicts of interest

There are no conflicts to declare.

## Supplementary Material
